# Human health risk and remedial goal assessment using Monte Carlo simulation: a case study of arsenic contaminated allotment gardens in southern Poland

**DOI:** 10.1007/s10653-026-03462-8

**Published:** 2026-09-04

**Authors:** Kayode Olabode, Karolina Lewińska, Izabela Komorowicz

**Affiliations:** 1 Faculty of Geographical and Geological Sciences, Adam Mickiewicz University, Krygowskiego 12, 61-680 Poznań, Poland; 2 Faculty of Chemistry, Adam Mickiewicz University, Uniwersytetu Poznańskiego 8, 61-614 Poznań, Poland

**Keywords:** In vitro bioaccessibility, Incidental soil ingestion, Historical mining, Simplified bioaccessibility extraction test, Dermal contact, Inhalation

## Abstract

This study quantified total arsenic (As), gastric-phase in vitro bioaccessible As (IVBA), and associated direct-soil-exposure risks for children and adults in mining-affected allotment gardens in Złoty Stok, Poland. Deterministic calculations and Monte Carlo simulations were applied to incidental soil ingestion, dermal contact, and inhalation of resuspended particles. Total As ranged from 433 to 1148 mgkg⁻^1^, while IVBA ranged from 4.5 to 23.5%. Under the total-As multi-pathway scenario, median hazard indices were 5.50 for children and 0.62 for adults. The 5th (P05) and 95th (P95) percentiles are 2.26 and 15.18 for children and 0.29 and 1.89 for adults. Median total cancer risk (CR_total_) was 1.78 × 10⁻^4^ for children and 1.06 × 10⁻^4^ for adults. Also, 80.0% of simulated child profiles and 54.0% of adult profiles produced CR_total_ estimates at or above 10⁻^4^. Deterministic dose and risk estimate generally fell between the probabilistic medians and P95 values. Applying IVBA only to the ingestion pathway reduced the median ingestion-related hazard quotient and cancer risk by 89.3%. Ingestion and dermal contact accounted for nearly all modeled risk, whereas inhalation contributed negligibly. Partial rank correlation analysis identified total soil As and soil ingestion rate as the principal positive risk drivers, while body weight was inversely associated with risk. At a target cancer risk of 10⁻^5^, the conservative child P05 remedial goal increased from 13.0 mgkg⁻^1^ under the total-As model to 34.8 mgkg⁻^1^ after IVBA adjustment of ingestion. The Polish soil-quality value of 20 mgkg⁻^1^ therefore lies between these two estimates, but both remedial goals remain far below the measured soil As concentrations, indicating that substantial reduction of direct soil exposure would be required across the study area.

**Supplementary Information:**

The online version contains supplementary material available at https://doi.org/10.1007/s10653-026-03462-8.

## Introduction

Arsenic (As) is a global toxicant of concern and is considered ubiquitous in the environment. It is a highly prevalent and toxic pollutant, and it has consistently ranked as a top priority on the Agency for Toxic Substances and Disease Registry’s substance priority list (ATSDR, 2023). As toxicity depends strongly on chemical form, dose, and exposure duration, thus inorganic As species generally present greater toxicological concern than most commonly encountered organic arsenicals (Cubadda et al., 2017; Yüksel et al., 2010, 2015). In fact, inorganic As species are classified as Group 1 human carcinogens (Cohen et al., 2016; IARC, 2004, 2012) and exposure has been associated with increased risk of skin, lung, and bladder cancers, including various developmental defects in children who experience early-life exposure through hand-to-mouth activity. (Palma-Lara et al., 2020; Vahter, 2008).

Centuries of gold (Au) and arsenic (As) mining in Złoty Stok, Poland, resulted in persistently elevated soil As concentrations (Karczewska et al., 2007; Muszer, 2011). According to Karczewska et al., (2007), up to 8800 mgkg^−1^ of As was observed in mine spoils, 17,200 mgkg^−1^ in tailings, and 11,500 mgkg^−1^ in alluvial soils in the Złoty Stok mine areas. In addition, elevated concentrations of As have also been reported in other areas of the town and in the surroundings (Lewińska & Karczewska, 2019, 2025). It is noteworthy that Złoty Stok features allotment gardens used for agricultural and recreational purposes. Extremely high concentrations of As have also been reported in these allotment gardens (Karczewska et al., 2010), exceeding the defined acceptable level in soils used for agricultural or recreational purposes in Poland (Rozporządzenie Ministra Środowiska, 2002) and some other European countries (Fendrich et al., 2024). Many residents rely on these gardens for vegetables and leisure gardening, thus creating a chronic exposure scenario. This underscores the need to assess the long-term risk posed by soil to humans, particularly children and gardeners who use it daily.

Gardeners and children face repeated contact with As-rich soil in allotment gardens through typical activities such as digging, planting, playing, and tending crops. Exposure routes include incidental ingestion of soil (ingestion), inhalation of fine dust, and dermal contact with soil (Ghezzi et al., 2023; Irwin et al., 2025; Loukola-Ruskeeniemi et al., 2022; Öncü et al., 2025). Ingestion clearly constitutes the dominant route of exposure (Lupolt et al., 2022). Children may experience comparatively high soil exposure because of frequent ground contact and hand-to-mouth behaviour. Compared with adults per kilogram of body weight, children eat, breathe, and drink more, making them more vulnerable to exposure. (Carroquino et al., 2012; Hauptman & Woolf, 2017). Therefore, conducting a human health risk assessment (HHRA) for such contaminated sites is a significant public health priority (Bhat et al., 2024; Emumejaye et al., 2025). Allotment garden settings also create potential food-chain exposure; however, the present study focuses specifically on direct soil contact.

HHRA compares estimated contaminant exposures with route-specific toxicity benchmarks to support contaminated-site evaluation and risk management (Ashbolt et al., 2013; Bień et al., 2005; Zhang et al., 2023). Soil assessments may include incidental ingestion, dermal contact, and inhalation of resuspended particles, although their relative contributions depend on receptor characteristics, exposure assumptions, and contaminant-specific toxicity values (Madear et al., 2020; Petruzzelli et al., 2025; Zhou et al., 2024). Deterministic assessment uses selected fixed values for contaminant concentration and exposure factors and therefore produces a single scenario-specific estimate. Depending on the selected inputs, this estimate may represent central-tendency, reasonable maximum exposure, or another defined condition. (Datta & Sarkar, 2005; Gong et al., 2022; Jha et al., 2023; Li et al., 2019; Moghadam et al., 2024; Rahman et al., 2023). Monte Carlo simulation (MCS) complements this approach by repeatedly sampling from assigned input distributions to generate a distribution of risk estimates. The resulting outputs can be summarized using central and upper-tail percentiles, benchmark-exceedance frequencies, and sensitivity measures that identify influential model inputs. MCS has increasingly been applied to probabilistic and source-specific health-risk assessments of contaminated soils (Miletić et al., 2025, 2024) Although MCS is well established in contaminated-soil assessment, few studies have combined paired measurements of total and gastric-phase bioaccessible As with route-specific multi-pathway risk calculations while preserving the measured relationship between total As and IVBA during probabilistic resampling. The present study addresses this gap in a mining-affected Polish allotment-garden setting.

Therefore, this study: (i) characterizes total As, and in vitro bioaccessible As (IVBA); (ii) compares deterministic and probabilistic total-As multi-pathway risks for children and adults; (iii) quantifies the effect of IVBA in a paired ingestion refinement; (iv) identifies influential model inputs using partial rank correlation coefficients; and (v) derives preliminary direct-soil-exposure remedial goal (RG) for several cancer-risk targets. A joint paired smoothed empirical bootstrap was used to preserve the measured relationship between total As and IVBA during probabilistic resampling.

## Materials and methods

### Study area and soil sampling

**Fig. 1:**
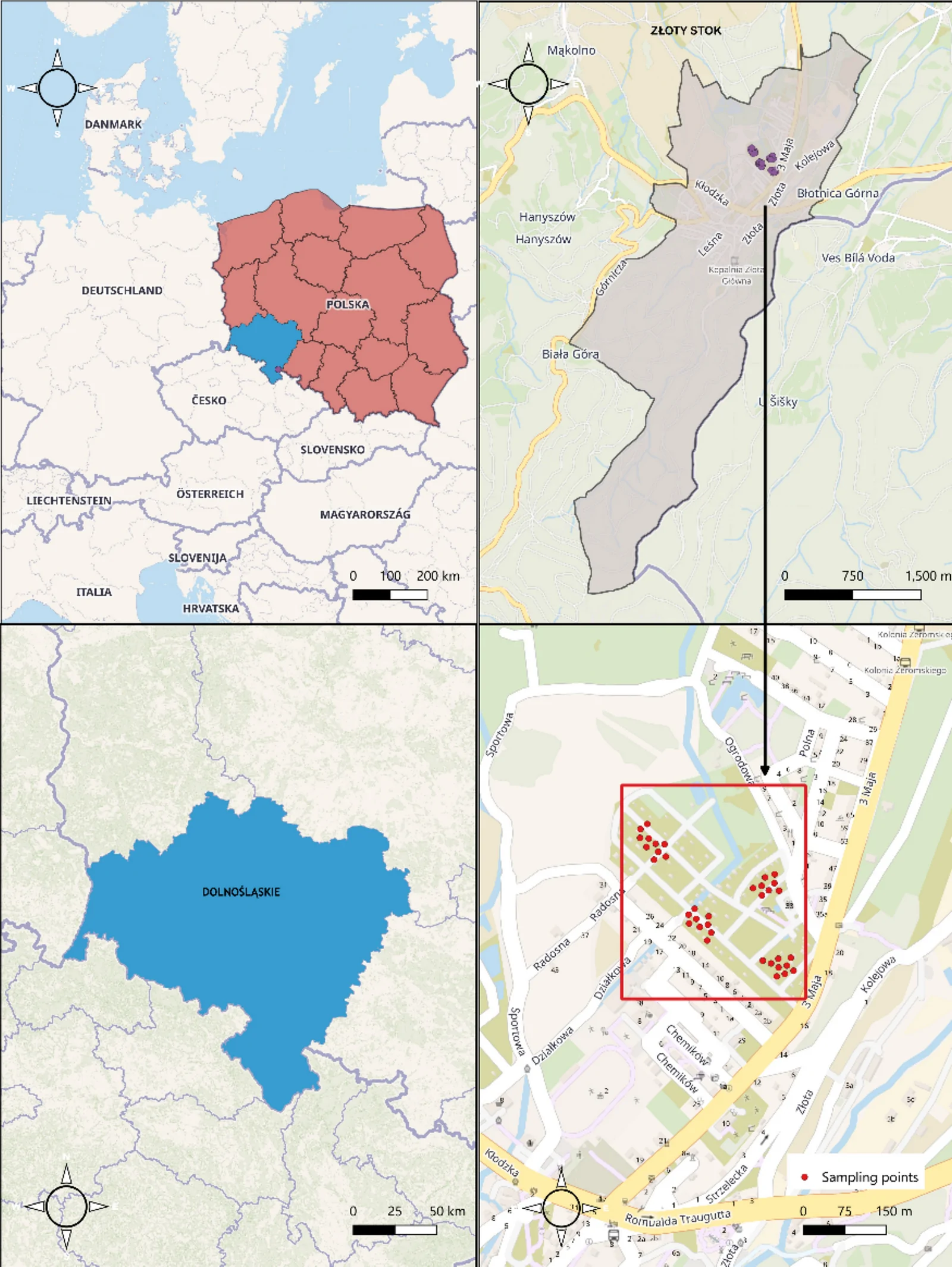
Location of the study area and distribution of soil-sampling points. **a** Poland in Central Europe, with the Lower Silesian Voivodeship highlighted; **b** Lower Silesian Voivodeship in southwestern Poland; **c** Złoty Stok and the location of the Radość allotment-garden complex; and **d** soil-sampling points within the Radość allotment-garden complex. Red dots indicate sampling locations, and the red rectangle delineates the sampled area

The study was conducted in the Radość allotment-garden complex in Złoty Stok, Lower Silesian, southwestern Poland (50° 26′ 47″ N, 16° 52′ 59″ E). The town is a former Au mining and ore-processing center where historical extraction, roasting, and disposal of As-bearing residues contributed to persistent enrichment of surrounding soils. (Muszer, 2011). Au mining is frequently associated with As-bearing minerals, and the latter is released into the surrounding environmental matrices during the extraction and processing (Besedin et al., 2023; Eisler, 2004; Faria et al., 2023; Mensah et al., 2020; Souza Neto et al., 2020; Straskraba & Moran, 1990). Thirty-six surface-soil samples were collected from 0–20 cm following the general sampling principles described by Zorzi et al., (2005). The geographical location of Złoty Stok and the distribution of the sampling points are presented in Fig. 1.

### Sample preparation and chemical analysis

Prior to chemical analysis, samples were air-dried, homogenized, and passed through a 2-mm sieve. A < 250 µm fraction was used for total As determination and IVBA testing because particles within this size range are more likely to adhere to hands and be incidentally ingested.

Total As in soil was determined using microwave-assisted digestion (Mars 6, CEM Corporation, Austria) followed by inductively coupled plasma mass spectrometry (ICP-MS). 0.2 g of soil aliquot was weighed directly into Teflon digestion vessels, after which 6 ml of 30% HCl (Suprapur, Merck, Darmstadt, Germany) and 2 ml of 65% HNO3 (Suprapur, Merck, Darmstadt, Germany) were added. After digestion, the suspensions were filtered through a 0.45 μm Whatman paper filter. The filtrate was transferred into 50 ml sample tubes and diluted to a final volume of 50 mL with Milli-Q deionized water. Samples were further diluted (× 25) before analysis for total As using ICP-MS (PlasmaQuant® MS Q, Analytik Jena, Germany).

In vitro bioaccessibility (IVBA) assays were performed on < 250 µm-sieved samples using the Simplified Bioaccessibility Extraction Test (SBET) method with a validated in vivo-in vitro correlation using mouse and swine models (Bradham et al., 2011, 2018). The SBET procedure is based on the USEPA 1340 soil extraction method, using an extraction solution of 0.4 M glycine adjusted to pH 1.50 ± 0.05 with dropwise addition of 6 M Suprapur HCl. This extraction solution was prepared to simulate human gastric juice (Billmann et al., 2023; USEPA, 2015a). The soil-solvent mixture (1:100) was extracted into a high-density polyethylene sample tube by incubation at 37 °C on an end-over-end rotator shaker at 30 rpm for 1 h. Before filtering the supernatant through a 0.45 µm Whatman filter, the sediment was allowed to settle. As the final step, the pH of each final filtrate was checked.

1 $${\text{IVBA (\% ) = }}\frac{{{\text{Bioaccessible As (mgkg}}^{ - 1} {) }}}{{{\text{Total As (mgkg}}^{ - 1} {)}}}{\text{ x 100}}$$

Instrument optimization and performance verification were conducted before each analytical sequence to ensure stable plasma conditions, efficient ion transmission, and adequate control of spectral interferences. The ICP–MS was operated at a radiofrequency power of 1.20 kW. The plasma, auxiliary, and nebulizer gas flow rates were maintained at 9.0, 1.5, and 1.02 L min^−1^, respectively. Bismuth, iridium, lithium, rhodium, scandium, and yttrium were used as internal standards to correct for instrumental drift and matrix-related variations in signal intensity. Data acquisition was performed using 20 scans for each of five replicate measurements. Measurements were conducted in both no-gas mode and integrated Collision Reaction Cell (iCRC) mode. Hydrogen was used as the reaction gas, while helium was used as the collision gas to reduce polyatomic interferences during elemental determination. These operating conditions were selected to provide stable signal response, adequate sensitivity and reliable correction of matrix and spectral effects during ICP–MS analysis.

Quality control and quality assurance procedures were implemented throughout sample preparation and instrumental analysis to ensure data accuracy and reliability. Procedural blanks (n = 8) and NIST SRM 2709a (San Joaquin Soil) were analyzed throughout the analytical sequence. The method limits of detection (LOD) and quantification (LOQ) were calculated as 3.3σ/S and 10σ/S, respectively, where σ represents the standard deviation of the procedural blank measurements, and S is the slope of the calibration curve. Based on the blank variability and calibration performance, the resulting method LOD and LOQ were 0.06 and 0.18 mgkg^−1^, respectively. All measured As concentrations were substantially higher than the method LOQ, indicating that the reported concentrations were quantified with adequate analytical confidence. Certified reference material (CRM) aliquots (n = 8) analyzed throughout the analytical sequence yielded a recovery of 95.7% after applying the corresponding calibration, procedural blank, and dilution corrections. The recovery was close to 100%, demonstrating satisfactory analytical trueness for the determination of As in the soil matrix. Potential outliers among the CRM measurements were evaluated using Dixon’s Q test at the 95% confidence level. No statistically significant outliers were identified; therefore, all eight measurements were retained. Instrumental data acquisition consisted of 20 scans for each of five replicate measurements, with internal standards used to correct for instrumental drift and matrix-related variation.

### Human health risk assessment and remedial goal

HHRA was evaluated for children and adults through incidental soil ingestion, dermal contact, and inhalation of resuspended particles. The primary assessment used total As for all pathways. A paired ingestion refinement compared total As ingestion with IVBA-adjusted ingestion. Identical environmental and exposure-factor draws were used within each MCS pair so that differences were attributable to the concentration basis. The recommended model for the assessment and quantification of human exposure and risk to soil As was applied following USEPA (2011). Human health risks were evaluated separately for children and adults via incidental soil ingestion, dermal contact, and inhalation of resuspended soil particles, using route-specific exposure equations commonly used in recent probabilistic assessments of potentially toxic elements (PTEs) in soils (Zhang et al., 2025).

Route-specific average daily dose (ADD; mgkg^−1^ day^−1^) for non-cancer assessment were calculated as:

2 $${\mathrm{ADD}}_{{{\mathrm{ing}}}} {\text{~ = ~}}\frac{{{\mathrm{C}}_{{{\mathrm{soil}}}} {\mathrm{~x~IR}}_{{{\mathrm{ing}}}} {\mathrm{~x~EF~x~ED~}}}}{{{\mathrm{BW~x~AT}}_{{{\mathrm{nc}}}} }}{\mathrm{~x~CF}}$$

3 $${\mathrm{ADD}}_{{{\mathrm{derm}}}} {\text{~ = ~}}\frac{{{\mathrm{C}}_{{{\mathrm{soil~}}}} {\mathrm{x~SA~x~AF~x~ABSd~x~EF~x~ED~}}}}{{{\mathrm{BW~x~AT}}_{{{\mathrm{nc}}}} }}{\mathrm{~x~CF}}$$

4 $${\mathrm{ADD}}_{{{\mathrm{inh}}}} {\text{~ = ~}}\frac{{{\mathrm{C}}_{{{\mathrm{soil}}}} {\mathrm{~x~IR}}_{{{\mathrm{inh}}}} {\mathrm{~x~EF~x~ED~}}}}{{{\mathrm{PEF~x~BW~x~AT}}_{{{\mathrm{nc}}}} }}$$

5 $${\mathrm{AT}}_{{{\mathrm{nc}}}} {\text{~ = ~ED~x~365}}$$

C_soil_ is the total As concentration in soil; IR_ing_ is soil ingestion rate; EF is exposure frequency; ED is exposure duration; CF is conversion factor (10^–6^ kgmg^−1^); BW is body weight; SA is exposed skin area; AF is soil-to-skin adherence; ABS_d_ is the dermal absorption fraction; IR_inh_ is inhalation rate; PEF is the particulate emission factor, and AT_nc_ is the non-cancer averaging time. CF was applied only to the ingestion and dermal-contact equations, not to inhalation, because dividing the soil As concentration by the PEF already yields the corresponding airborne concentration.

The hazard quotient (HQ), which is the non-carcinogenic risk estimate, is calculated for each exposure route: ingestion (HQ_ing_), dermal contact (HQ_derm_), and inhalation (HQ_inh_). Hazard index (HI) is the summation of the non-carcinogenic risk from the exposure pathways.

6 $${\mathrm{HQ}}_{{{\mathrm{ing}}}} {\text{~ = ~}}\frac{{{\mathrm{ADD}}_{{{\mathrm{ing}}}} {\mathrm{~}}}}{{{\mathrm{RfD}}_{{{\mathrm{ing}}}} }}$$

7 $${\mathrm{HQ}}_{{{\mathrm{derm}}}} {\text{~ = ~}}\frac{{{\mathrm{ADD}}_{{{\mathrm{derm}}}} {\mathrm{~}}}}{{{\mathrm{RfD}}_{{{\mathrm{derm}}}} }}$$

8 $${\mathrm{HQ}}_{{{\mathrm{inh}}}} {\text{~ = ~}}\frac{{{\mathrm{ADD}}_{{{\mathrm{inh}}}} {\mathrm{~}}}}{{{\mathrm{RfD}}_{{{\mathrm{inh}}}} }}$$

9 $${\mathrm{HI}} = {\mathrm{HQ}}_{{{\mathrm{ing}}}} ~ + ~{\mathrm{HQ}}_{{{\mathrm{derm}}}} ~ + ~{\mathrm{HQ}}_{{{\mathrm{inh}}}}$$

HQ ≤ 1 indicates that non-cancer effects are unlikely and can be considered negligible, whereas HQ > 1 suggests that the estimated exposure exceeds the reference dose (RfD). However, HQ > 1 does not imply a statistically significant probability of adverse health effects. Hazard quotients are not probabilistic measures; therefore, neither the likelihood nor the severity of health effects increases linearly as HQ approaches or exceeds the RfD. (Imsilp et al., 2024; Miletić et al., 2025; USEPA, 2015b). RfD values are presented in Table 1.

**Table 1:** Probability distributions and values used for probabilistic assessment of risk to human health

Parameter	Unit	Child	Adult	Distributions	Source
Total soil As concentration	mgkg^−1^	698.08	698.08	Joint paired smoothed empirical resampling	Measured study data; Augustsson et al., (2015); USEPA, (1997)
IVBA fraction	Fraction	0.1045	0.1045	Jointly resampled with total As	Measured study data; Augustsson et al., (2015); USEPA, (1997)
Soil ingestion rate	mg day^−1^	200	100	Lognormal	Karimian et al., (2021); Stanek and Calabrese, (2000)
Exposure frequency	Days year^−1^	144	144	Triangular	Miletić et al., (2023); Różański et al., (2021)
Body weight	kg	16.8	70	Lognormal	Różański et al., (2021); USEPA, (2015a, b, c, d)
Skin surface area	cm^2^	2800	5700	Lognormal	Karimian et al., (2021)
Soil adherence factor	mg cm^−2^event^−1^	0.20	0.07	Fixed	USEPA, (2015a, b, c, d)
Dermal absorption fraction		0.03	0.03	Fixed	USEPA, (2015a, b, c, d)
Inhalation rate	m^3^ day^−1^	10	15	Lognormal	Harris et al., (1996); Miletić et al., (2023); USEPA, (2002)
Exposure duration	Years	6	30	Lognormal	Smith, (1994); USEPA, (2002)
Particle emission factor	m^3^ kg^−1^	1.36 × 10⁹	1.36 × 10⁹	Fixed	USEPA, (2015d)
Soil-mass conversion factor	kg mg^−1^	1 × 10⁻⁶	1 × 10⁻⁶	Fixed	–
Lifetime	Years	78	78	Fixed	GUS, (2025)
Non-cancer averaging time	Days	ED × 365	ED × 365	Fixed	Zhou et al., (2024)
Cancer averaging time	Days	78 × 365	78 × 365	Fixed	Zhou et al., (2024)
Ingestion RfD	mgkg^−1^ day^−1^	3.00 × 10⁻^4^	3.00 × 10⁻^4^	Fixed	López et al., (2025)
Dermal RfD	mgkg^−1^ day^−1^	1.23 × 10⁻^4^	1.23 × 10⁻^4^	Fixed	Wang et al., (2025)
Inhalation RfD	mgkg^−1^ day^−1^	1.23 × 10⁻^4^	1.23 × 10⁻^4^	Fixed	Wang et al., (2025)
Ingestion cancer slope factor	(mgkg^−1^ day^−1^)^−1^	1.50	1.50	Fixed	Wang et al., (2025)
Dermal cancer slope factor	(mgkg^−1^ day^−1^)^−1^	3.66	3.66	Fixed	Wang et al., (2025)
Inhalation cancer slope factor	(mgkg^−1^ day^−1^)^−1^	1.51	1.51	Fixed	Wang et al., (2025)

For cancer effects, route-specific lifetime average daily dose (LADD; mgkg^−1^ day^−1^) were calculated using a lifetime averaging time before application of the corresponding slope factors:

10 $${\mathrm{LADD}}_{{{\mathrm{ing}}}} {\text{~ = ~}}\frac{{{\mathrm{C}}_{{{\mathrm{soil}}}} {\mathrm{~x~IR}}_{{{\mathrm{ing}}}} {\mathrm{~x~EF~x~ED~}}}}{{{\mathrm{BW~x~AT}}_{{{\mathrm{ca}}}} }}{\mathrm{~x~CF}}$$

11 $${\mathrm{LADD}}_{{{\mathrm{derm}}}} {\text{~ = ~}}\frac{{{\mathrm{C}}_{{{\mathrm{soil}}}} {\mathrm{~x~SA~x~AF~x~ABSd~x~EF~x~ED~}}}}{{{\mathrm{BW~x~AT}}_{{{\mathrm{ca}}}} }}{\mathrm{~x~CF}}$$

12 $${\mathrm{LADD}}_{{{\mathrm{inh}}}} {\text{~ = ~}}\frac{{{\mathrm{C}}_{{{\mathrm{soil}}}} {\mathrm{~x~IR}}_{{{\mathrm{inh}}}} {\mathrm{~x~EF~x~ED~}}}}{{{\mathrm{PEF~x~BW~x~AT}}_{{{\mathrm{ca}}}} }}$$

13 $${\mathrm{AT}}_{{{\mathrm{ca}}}} {\text{~ = ~LT~x~365}}$$

AT_ca_ is the cancer averaging time.

14 $${\mathrm{CR}}_{{{\mathrm{ing}}}} = {\mathrm{LADD}}_{{{\mathrm{ing}}}} {\mathrm{~x~SF}}_{{{\mathrm{ing}}}}$$

15 $${\mathrm{CR}}_{{{\mathrm{derm}}}} = {\mathrm{LADD}}_{{{\mathrm{derm}}}} {\mathrm{~x~SF}}_{{{\mathrm{derm}}}}$$

16 $${\mathrm{CR}}_{{{\mathrm{inh}}}} = {\mathrm{LADD}}_{{{\mathrm{inh}}}} {\mathrm{~x~SF}}_{{{\mathrm{inh}}}}$$

17 $${\mathrm{CR}}_{{{\mathrm{total}}}} = {\mathrm{CR}}_{{{\mathrm{ing}}}} {\mathrm{~}} + {\mathrm{~CR}}_{{{\mathrm{derm}}}} {\mathrm{~}} + {\mathrm{~CR}}_{{{\mathrm{inh}}}}$$

CR value below 10^–6^ suggests a negligible carcinogenic risk, > 10^–4^ is unacceptable, and from 10^–6^ to 10^–4^ is considered acceptable risk (Demissie et al., 2024; USEPA, 2015b; Vesković & Onjia, 2025a). HI and CR_total_ were obtained by summing the corresponding route-specific estimates, consistent with the multi-pathway probabilistic soil-risk assessments (Zhang et al., 2025).

A secondary paired analysis was conducted for the ingestion pathway using IVBA-adjusted As concentrations as follows;

18 $${\mathrm{C}}_{{{\mathrm{ing}}{\mathrm{.IVBA}}}} = {\mathrm{C}}_{{{\mathrm{total}}}} {\mathrm{~x~f}}_{{{\mathrm{IVBA}}}}$$

19 $${\mathrm{HQ}}_{{{\mathrm{ing,IVBA}}}} {\text{~ = ~}}\frac{{{\mathrm{ADD}}_{{{\mathrm{ing,IVBA}}}} {\mathrm{~}}}}{{{\mathrm{RfD}}_{{{\mathrm{oral}}}} }}$$

20 $${\mathrm{CR}}_{{{\mathrm{ing,IVBA}}}} = {\mathrm{LADD}}_{{{\mathrm{ing,IVBA}}}} {\mathrm{~x~SF}}_{{{\mathrm{ing}}}}$$

where C_ing,IVBA_ is the IVBA-adjusted ingestion concentration, C_total_ is the total soil As concentration, f_IVBA_ is the paired IVBA expressed as a decimal fraction, ADD_ing,IVBA_ and LADD_ing,IVBA_ are the corresponding average daily dose and lifetime average daily dose, respectively, RfD_ing_ is the oral reference dose, and SF_ing_ is the oral cancer slope factor for inorganic As.

RG was calculated for target lifetime CR of 10^–6^, 10^–5^, and 10^–4^ (USEPA, 1991). For each pathway, a unit concentration CR coefficient was calculated from the relevant exposure factors and route-specific slope factor.

For each exposure pathway j:

21 $${\mathrm{CR}}_{{\mathrm{j}}} = {\mathrm{CR}}_{{{\mathrm{soil}}}} {\mathrm{~x~K}}_{{\mathrm{j}}} {\mathrm{~}}$$

where K_j_ is the route-specific cancer risk coefficient per unit increase in soil As concentration.

The route-specific RG is:

22 $${\mathrm{RG}}_{{\mathrm{j}}} {\text{ = ~}}\frac{{{\mathrm{TR~}}}}{{{\mathrm{K}}_{{\mathrm{j}}} }}$$

For the total-As multi-pathway scenario:

23 $${\mathrm{K}}_{{{\mathrm{total}}}} = {\mathrm{K}}_{{{\mathrm{ing}}}} + {\mathrm{~K}}_{{{\mathrm{derm}}}} {\mathrm{~}} + ~{\mathrm{K}}_{{{\mathrm{inh}}}}$$

24 $${\mathrm{RG}}_{{{\mathrm{total}}}} {\text{ = ~}}\frac{{{\mathrm{TR~}}}}{{{\mathrm{K}}_{{{\mathrm{ing}}}} + {\mathrm{~K}}_{{{\mathrm{derm}}}} {\mathrm{~}} + ~{\mathrm{K}}_{{{\mathrm{inh}}}} }}$$

For the IVBA-adjusted scenario, IVBA was applied only to ingestion:

25 $${\mathrm{RG}}_{{{\mathrm{total,IVBA}}}} {\text{ = ~}}\frac{{{\mathrm{TR~}}}}{{{\mathrm{f}}_{{{\mathrm{IVBA}}}} {\mathrm{K}}_{{{\mathrm{ing}}}} + {\mathrm{~K}}_{{{\mathrm{derm}}}} {\mathrm{~}} + ~{\mathrm{K}}_{{{\mathrm{inh}}}} }}$$

where TR is the target risk, f_IVBA_ is IVBA expressed as a decimal fraction and K_ing,_ K_derm_, and K_inh_ are the ingestion, dermal contact, and inhalation cancer-risk coefficients per unit soil concentration. RG was calculated for every MCS iteration and summarized by P05, median, mean, and P95. Given that risk per unit of concentration increases due to pathway summation, the total multi-pathway RG is necessarily lower than the ingestion-only RG when all pathway coefficients are positive. RG values were interpreted as preliminary direct soil exposure concentrations rather than enforceable cleanup standards.

### Monte Carlo simulation

Probabilistic HHRA was performed separately for child and adult receptors using a one-dimensional Monte Carlo simulation (MCS) in RStudio 2026.01.0+392 “Apple Blossom” Release. Recent studies have applied probabilistic MCS to contaminant-related groundwater risk assessment and have extended the approach to two-dimensional MCS integrated with source analysis and spatial risk mapping (Vesković & Onjia, 2025a, b). The present study retained a one-dimensional structure because As concentrations and receptor variability were propagated under fixed distribution forms and parameters. Parameter uncertainty was not modeled in a separate simulation level. The deterministic inputs, probabilistic distributions, and distribution parameters used in the model are summarized in Table 1. For each receptor and model scenario, 200,000 iterations were generated. In each iteration, a paired total-As and IVBA observation was produced using joint smoothed empirical resampling. Total As was transformed using the natural logarithm, and IVBA using the logit function. A measured pair was sampled with replacement, a correlated Gaussian-kernel perturbation was applied on the transformed scale, and the values were constrained to the observed transformed ranges before back-transformation. Bioaccessible As was then calculated as the product of total As and IVBA. This procedure generated continuous input distributions while preserving the measured dependence between total As and IVBA. Receptor-specific exposure factors were sampled from their assigned distributions at every iteration. The sampled As concentration and exposure inputs were entered into the route-specific dose and risk equations to calculate ADD, LADD, HQ, and CR for ingestion, dermal contact, and inhalation, after which the pathway-specific values were summed to obtain total HI and CR_total_. For the paired ingestion comparison, the total-As and IVBA-adjusted calculations used identical records and exposure-factor draws within each iteration except for the ingestion concentration basis. This pairing isolated the effect of IVBA adjustment from variation in the other model inputs. Random-number generation used the L’Ecuyer-CMRG algorithm with a master seed of 12,345 to permit reproducibility. Probabilistic outputs were summarized using the 5th percentile (P05), median, mean and 95th percentile (P95), together with the proportions exceeding the selected risk benchmarks. Numerical convergence was evaluated progressively at 1,000, 5000, 10,000, 25,000, 50,000, 100,000 and 200,000 iterations by examining the stability of the mean, median, and P95 of HI and CR_total_. The As concentrations resampling configuration, measured-versus-simulated diagnostics, total As–IVBA dependence, distributional tests, receptor-input diagnostics and convergence assessment are reported in Tables S2–S9 and Figure S1 of the supplementary information.

## Results and discussion

### Soil As concentrations and bioaccessibility

Table 2 presents the As concentrations in soils. total As ranged from 433 to 1148 mgkg^−1^. The mean total As concentration was 698 ± 237 mgkg^−1^ with a coefficient of variation (CV) of 34%. These levels of As are ~ 35 times above the maximum allowable concentration (20 mgkg^−1^) in Polish agricultural soils (Rozporządzenie Ministra Środowiska, 2002), thus implying serious environmental concern. Previous studies in Złoty Stok have also reported extremely high As concentrations in the soils. For example, Stachnik et al. (2020) reported up to 1000 mgkg^−1^ in Quaternary alluvial sediments around Złoty Stok. In fact, up to 8800 mgkg^−1^ has been reported in the Złoty Stok mine spoil by Karczewska et al. (2007). Furthermore, Karczewska et al. (2010) recorded up to 250 mgkg^−1^ in allotment gardens. Soil As concentrations < 100 mgkg^−1^ have been reported in many vegetable gardens (Mathee et al., 2018; McHale et al., 2025; Paltseva et al., 2018). However, much higher As levels have been found in some garden soils. For example, 374 mgkg^−1^ of As was reported by Ramirez-Andreotta et al. (2013) in mining-affected home garden soils in Arizona, USA. Vejvodová et al. (2022) also reported 418 mgkg^−1^ of As from the surface soils of vegetable garden soils collected from the vicinity of Kutná Hora, Czechia. Thus, these concentrations were at the 5th percentile (436 mgkg^−1^) value of the soil As concentrations determined in this study, thereby highlighting a high level of human As exposure in Złoty Stok allotment gardens. These might be due to the weathering of arsenopyrite and other As-bearing primary minerals, which can release As into pore water, where it may be retained through adsorption or coprecipitation with Fe and Al oxyhydroxides, or transformed into secondary arsenate minerals (Rajpert et al., 2016; Stachnik et al., 2020). Furthermore, Tokatlı et al. (2025) demonstrated that concentrations of PTEs and associated risks may vary considerably across locations and seasons, highlighting the importance of spatial and temporal context.

**Table 2:** Descriptive statistics for measured total arsenic, bioaccessible arsenic, and in vitro bioaccessibility

Variable	Unit	Minimum	P05	Median	Mean	SD	P95	Maximum	CV (%)
Total As	mgkg⁻^1^	433	435	595	698	237	1057	1148	34
Bioaccessible As	mgkg⁻^1^	48.05	50.37	62.90	72.94	23.24	115	135	32
IVBA	%	4.5	5.5	10.7	11.1	3.7	20.6	23.5	34

The minimum, 5th percentile, median, arithmetic mean, standard deviation, 95th percentile, maximum values and coefficient of variation for total soil As, bioaccessible As and IVBA are presented (n = 36)

IVBA in the tested soil ranged from 5 to 23%. The recorded IVBA mean value was 11 ± 3.72% with a CV of 34%. This is an important refinement in the HHRA as it is the fraction that is solubilized under in vitro gastric conditions and potentially available for intestinal absorption. The contrast between a very high total As and a low IVBA indicates that total concentration alone does not describe the fraction potentially released under gastric conditions. This indicates that As in the contaminated soils was mostly associated with less soluble solid mineral phases, which were not released during the acidic gastric extraction (Billmann et al., 2023; McHale et al., 2025). Previous studies of mining-affected soils have linked bioaccessible As particularly to exchangeable, surface-bound, and amorphous Fe-associated fractions, while increasing Fe-oxide crystallinity and mineral stability reduce gastric release (Ehlert et al., 2018; Fazle Bari et al., 2021; Palumbo-Roe et al., 2015). Drahota et al. (2025) reported bioaccessibility ranging from less than 1% for stable minerals such as scorodite and pharmacosiderite to near-complete release of readily soluble arsenates, thereby suggesting that IVBA depends largely on mineral identity. Therefore, using total As concentration in HHRA can lead to an overestimation of risk because the bioaccessible fraction of As is potentially available for intestinal absorption. However, many conventional HHRAs ignore this important factor and use total As in risk calculations, thereby assuming 100% bioaccessibility and overestimating the internal dose.

### Average daily dose and lifetime average daily dose

ADD of As via ingestion, dermal contact, and inhalation for children and adults is presented in Table 3. The deterministic ADD for children via the ingestion route was 3.28 × 10^–3^ mgkg^−1^ day^−1^, whereas that for adults was 3.93 × 10^–4^ mgkg^−1^ day^−1^. The corresponding LADD were 2.52 × 10^–4^ and 1.51 × 10^–4^ mgkg^−1^ day^−1^ for children and adults, respectively. However, the probabilistic 95th percentile (P95) ADD values for both children and adults are 3.59 × 10^–3^ mgkg^−1^ day^−1^ and 4.17 × 10^–4^ mgkg^−1^ day^−1^, respectively. Median LADD was 6.95 × 10^–5^ and 2.38 × 10^–5^ mgkg^−1^ day^−1^, while the P95 values were 2.95 × 10^–4^ and 1.61 × 10^–4^ mgkg^−1^ day^−1^. The closeness between the deterministic value and the 95th percentile value is a structural consequence of the right-skewed propagation of the input distributions. When the total As concentration with CV of 34% is combined with lognormal exposure factors, the resulting dose distribution concentrates probability mass toward its lower end. In addition, inhalation and dermal contact also contribute to the total ADD of both children and adults. The ADD of 2.75 × 10^–4^ and 1.21 × 10^–7^ mgkg^−1^ day^−1^ was estimated deterministically for children, whereas for adults, the values were 4.71 × 10^–5^ mgkg^−1^ day^−1^ and 4.34 × 10^–8^ mgkg^−1^ day^−1^, respectively. P95 estimates yielded 5.86 × 10^–4^ mgkg^−1^ day^−1^ and 2.37 × 10^–7^ mgkg^−1^ day^−1^ for children, while adults have a slightly lower ADD via both dermal contact and inhalation (8.33 10^–5^ mgkg^−1^ day^−1^ and 7.65 × 10^–8^ mgkg^−1^ day^−1^. Median dermal LADD was 1.72 × 10^−5^ mgkg^−1^ day^−1^ for children and 1.67 × 10^−5^ mgkg^−1^ day^−1^ for adults, with respective P95 values of 5.11 × 10^−5^ and 3.26 × 10^−5^ mgkg^−1^ day^−1^. Inhalation produced substantially smaller LADD, with median values of 7.80 × 10^−9^ and 1.53 × 10^−8^ mgkg^−1^ day^−1^ for children and adults, respectively. Although children have lower body weight than adults and adults have a larger exposed skin area, their median dermal LADDs are similar. This is because of the partial cancellation in which the child’s higher soil-to-skin adherence factor and lower body weight are balanced against the adult’s lower adherence factor and higher body weight, leaving the median dose essentially the same across receptor groups. Sander and Öberg (2006) reported a lower As total ADD using deterministic and probabilistic approaches in their assessment of a closed steel mill site in southern Sweden, where deterministic ADD values (9.2 × 10^–4^ and 4.3 × 10^–4^ mgkg^−1^ day^−1^, respectively) exceeded those obtained probabilistically. Similarly, Emumejaye et al., (2025) reported deterministic ADD values of 2.70 × 10^–4^ and 2.77 × 10^–4^ mgkg^−1^ day^−1^ for the ingestion, 6.69 × 10^–6^ and 3.20 × 10^–6^ mgkg^−1^ day^−1^ for dermal contact and 4.15 × 10^–9^ and 1.99 × 10^–9^ mgkg^−1^ day^−1^ for inhalation pathways respectively, in As-contaminated soils from a mining belt in Nigeria. In contrast, Mendoza-Lagunas et al., (2025) applied probabilistic modeling and they obtained lower ADD estimates for school-aged children of 9.39 × 10^–5^, 1.30 × 10^–4^, and 9.08 × 10^–9^ mgkg⁻^1^ day⁻^1^ for ingestion, dermal contact and inhalation pathways respectively and 1.68 × 10⁻^5^, 7.30 × 10^–5^, 5.69 × 10^–9^ mgkg⁻^1^ day⁻^1^ for adults in a community exposed to As-rich dust in Araró, Mexico. These values were generally lower than those observed in the present study, highlighting the severity of exposure conditions in Złoty Stok, particularly because the ADD for children is usually higher than that for adults, both deterministically and probabilistically. This pattern is well established in the literature, as children typically receive higher doses due to their soil intake relative to body mass, which is substantially greater than that of adults (Miletić et al., 2023). In this study, exposure via the ingestion route accounts for 83% of children’s ADD and 71% of adults’ ADD probabilistically. However, the percentage contribution of the ingestion exposure pathway to the ADD for children and adults was 92% and 89%, respectively, under the deterministic approach. Ingestion exposure is of particular concern, especially to children, as they often engage in hand-to-mouth activities Pujalté et al., 2025.

**Table 3:** Deterministic and probabilistic route-specific average daily doses and lifetime average daily doses for children and adults

Receptor	Dose metric	Deterministic	P05	Median	Mean	P95
Child	ADD_ing_	3.28 × 10⁻^3^	2.67 × 10⁻^4^	9.71 × 10⁻^4^	1.33 × 10⁻^3^	3.59 × 10⁻^3^
Child	ADD_derm_	2.75 × 10⁻^4^	1.03 × 10⁻^4^	2.4 × 10⁻^4^	2.8 × 10⁻^4^	5.86 × 10⁻^4^
Child	ADD_inh_	1.21 × 10⁻⁷	5.27 × 10⁻⁸	1.08 × 10⁻⁷	1.22 × 10⁻⁷	2.37 × 10⁻⁷
Child	LADD_ing_	2.52 × 10⁻^4^	1.65 × 10⁻^5^	6.95 × 10⁻^5^	1.02 × 10⁻^4^	2.95 × 10⁻^4^
Child	LADD_derm_	2.12 × 10⁻^5^	5.97 × 10⁻⁶	1.72 × 10⁻^5^	2.15 × 10⁻^5^	5.11 × 10⁻^5^
Child	LADD_inh_	9.27 × 10⁻⁹	2.95 × 10⁻⁹	7.8 × 10⁻⁹	9.39 × 10⁻⁹	2.12 × 10⁻⁸
Adult	ADD_ing_	3.93 × 10⁻^4^	9.31 × 10⁻⁶	6.19 × 10⁻^5^	1.22 × 10⁻^4^	4.17 × 10⁻^4^
Adult	ADD_derm_	4.71 × 10⁻^5^	2.61 × 10⁻^5^	4.33 × 10⁻^5^	4.84 × 10⁻^5^	8.33 × 10⁻^5^
Adult	ADD_inh_	4.34 × 10⁻⁸	2.42 × 10⁻⁸	3.98 × 10⁻⁸	4.46 × 10⁻⁸	7.65 × 10⁻⁸
Adult	LADD_ing_	1.51 × 10⁻^4^	3.54 × 10⁻⁶	2.38 × 10⁻^5^	4.67 × 10⁻^5^	1.61 × 10⁻^4^
Adult	LADD_derm_	1.81 × 10⁻^5^	9.75 × 10⁻⁶	1.67 × 10⁻^5^	1.86 × 10⁻^5^	3.26 × 10⁻^5^
Adult	LADD_inh_	1.67 × 10⁻⁸	9.03 × 10⁻⁹	1.53 × 10⁻⁸	1.72 × 10⁻⁸	2.99 × 10⁻⁸

Deterministic values represent fixed-point estimates calculated from the selected receptor-specific input values. Probabilistic results are summarized by the 5th percentile, median, arithmetic mean and 95th percentile. All values are in mgkg^−1^

### Hazard quotient and index

**Fig. 2:**
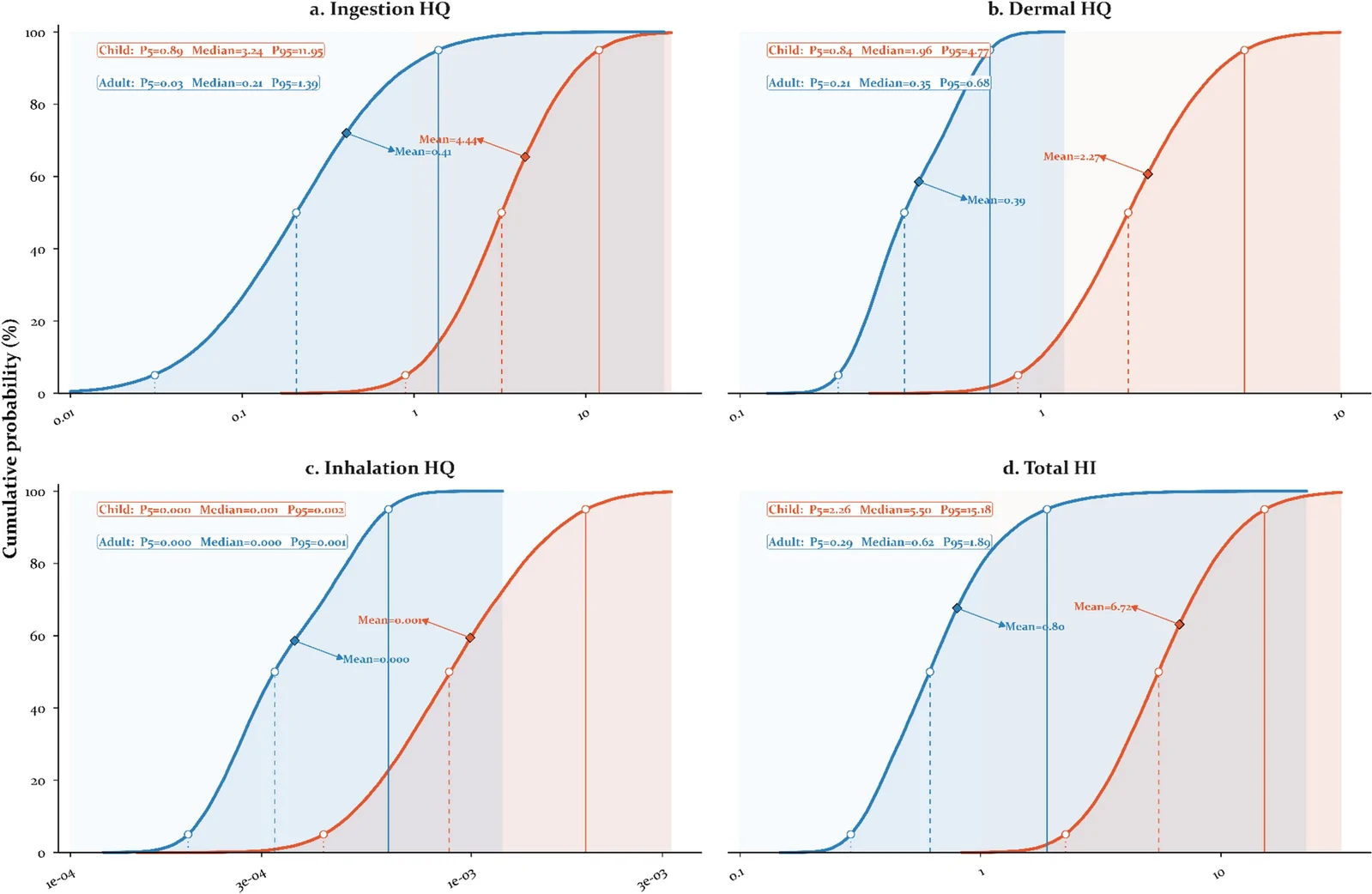
Cumulative probability distributions of HI for children and adults under the primary total As scenario: **a** HQ_ing_, **b** HQ_derm_, **c** HQ_inh_, and **d** HI. Distribution was summarized by the 5th percentile, median, mean, and 95th percentile. The reference value of 1 represents the screening benchmark and values exceeding 1 indicate the potential for non-cancer effects

The deterministic and probabilistic estimates of HQ and HI for both children and adults are presented in Table 4. Deterministic estimation of HQ_ing_ (non-carcinogenic adverse health effects) was 10.93 and 1.31 for children and adults, respectively. HQ_derm_ was 2.24 and 0.38, and HQ_inh_ was 9.80 × 10^–4^ and 3.53 × 10^–4^ for children and adults, respectively. HI for both children and adults was 13.17 and 1.70, respectively. These values were higher than those obtained by Gong et al. (2022) for agricultural soils in China, where HQ values for ingestion exposure were 0.39 for children and 0.05 for adults, and HI values were 0.39 and 0.06, respectively. In this study, the ingestion route of exposure is the primary contributor to children's HI with a probabilistic mean and P95 contributions of 60.7 and 86.3% respectively. (Table S11). This is consistent with the findings reported by Chen et al. (2022) in a study conducted on an agricultural soil adversely affected by Au mining activities in China. The deterministic estimates being lower than the corresponding probabilistic P95 and HI values is itself a structural consequence of the right-skewed input distribution. The fixed-input reasonable maximum exposure scenario gives a single point estimate that does not capture the breadth of the underlying dispersion; the probabilistic distribution, by contrast, summarizes the full range of plausible HQ across the simulated receptor population. Probabilistically, children’s P95  HQ values in this study were 11.96, 4.77, and 0.002 for ingestion, dermal contact, and inhalation pathways respectively, whereas those for adults are 1.39, 0.68, and 6.22 × 10^–4^. HI values were 15.18 and 1.89 for children and adults, respectively. Mendoza-Lagunas et al. (2025) reported significantly lower P95 HI estimates for children and adults (0.65 and 0.27, respectively). As determined in this study, probabilistic (P95) non-carcinogenic health effects estimates exceed deterministic estimates. Shen et al. (2019) found that, for both children and adults, HQ_ing_ values were 3.58 and 0.50, respectively, whereas HI values were 6.03 and 1.42, respectively. This is comparable with the higher estimates obtained in this study, as shown in Fig. 2. Across both receptor groups and exposure scenarios, the typical pattern (95th percentile > mean > 5th percentile) confirms the expected right-skewed input total As concentration and the lognormal exposure factors. The child HI median of 5.50 and the adult median of 0.62 anchor the central tendency well below the conservative deterministic point estimates, yet the child median already exceeds the 1 threshold. This distribution allows one to distinguish between the typical receptor exposure and the high-end tail in a way that a single deterministic value cannot. The inhabitants of these allotment gardens are therefore at risk of non-cancer health effects associated with As exposure, and children are ~ 15 times more susceptible than adults.

**Table 4:** Deterministic point estimates and probabilistic summaries of route-specific and total non-cancer and cancer risk under the primary total-arsenic model

Receptor	Risk	Deterministic	P05	Median	Mean	P95	Above benchmark (%)
Child	HQ_ing_	10.93	0.89	3.24	4.44	11.95	93.3
Child	HQ_derm_	2.24	0.84	1.96	2.27	4.77	90.2
Child	HQ_inh_	9.80 × 10⁻^4^	4.28 × 10⁻^4^	8.81 × 10⁻^4^	9.94 × 10⁻^4^	2.00 × 10⁻^3^	0.0
Child	HI	13.17	2.26	5.50	6.72	15.18	100
Adult	HQ_ing_	1.31	0.03	0.21	0.41	1.39	8.7
Adult	HQ_derm_	0.38	0.21	0.35	0.39	0.68	0.0
Adult	HQ_inh_	3.53 × 10⁻^4^	1.97 × 10⁻^4^	3.23 × 10⁻^4^	3.63 × 10⁻^4^	6.22 × 10⁻^4^	0.0
Adult	HI	1.70	0.29	0.62	0.80	1.89	20.7
Child	CR_ing_	3.78 × 10⁻^4^	2.47 × 10⁻^5^	1.04 × 10⁻^4^	1.54 × 10⁻^4^	4.43 × 10⁻^4^	51.9
Child	CR_derm_	7.75 × 10⁻^5^	2.18 × 10⁻^5^	6.31 × 10⁻^5^	7.86 × 10⁻^5^	1.87 × 10⁻^4^	24.5
Child	CR_inh_	1.40 × 10⁻⁸	4.45 × 10⁻⁹	1.18 × 10⁻⁸	1.42 × 10⁻⁸	3.20 × 10⁻⁸	0.0
Child	CR_total_	4.56 × 10⁻^4^	5.89 × 10⁻^5^	1.78 × 10⁻^4^	2.32 × 10⁻^4^	5.82 × 10⁻^4^	80.0
Adult	CR_ing_	2.27 × 10⁻^4^	5.31 × 10⁻⁶	3.56 × 10⁻^5^	7.01 × 10⁻^5^	2.41 × 10⁻^4^	18.7
Adult	CR_derm_	6.63 × 10⁻^5^	3.57 × 10⁻^5^	6.10 × 10⁻^5^	6.82 × 10⁻^5^	1.19 × 10⁻^4^	14.1
Adult	CR_inh_	2.52 × 10⁻⁸	1.36 × 10⁻⁸	2.32 × 10⁻⁸	2.59 × 10⁻⁸	4.52 × 10⁻⁸	0.0
Adult	CR_total_	2.93 × 10⁻^4^	4.88 × 10⁻^5^	1.06 × 10⁻^4^	1.38 × 10⁻^4^	3.28 × 10⁻^4^	54.0

Route-specific and total non-cancer and lifetime cancer-risk estimates for children and adults under the primary model, in which total soil As was used for ingestion, dermal contact, and inhalation. Deterministic results are fixed-point estimates, not means of probabilistic distributions. Probabilistic values are summarized by the 5th percentile, median, arithmetic mean, and 95th percentile. The percentage above the benchmark represents the frequency of simulated exposure profiles with HQ or HI > 1, or cancer risk ≥ 10^−4^

### Cancer risk

**Fig. 3:**
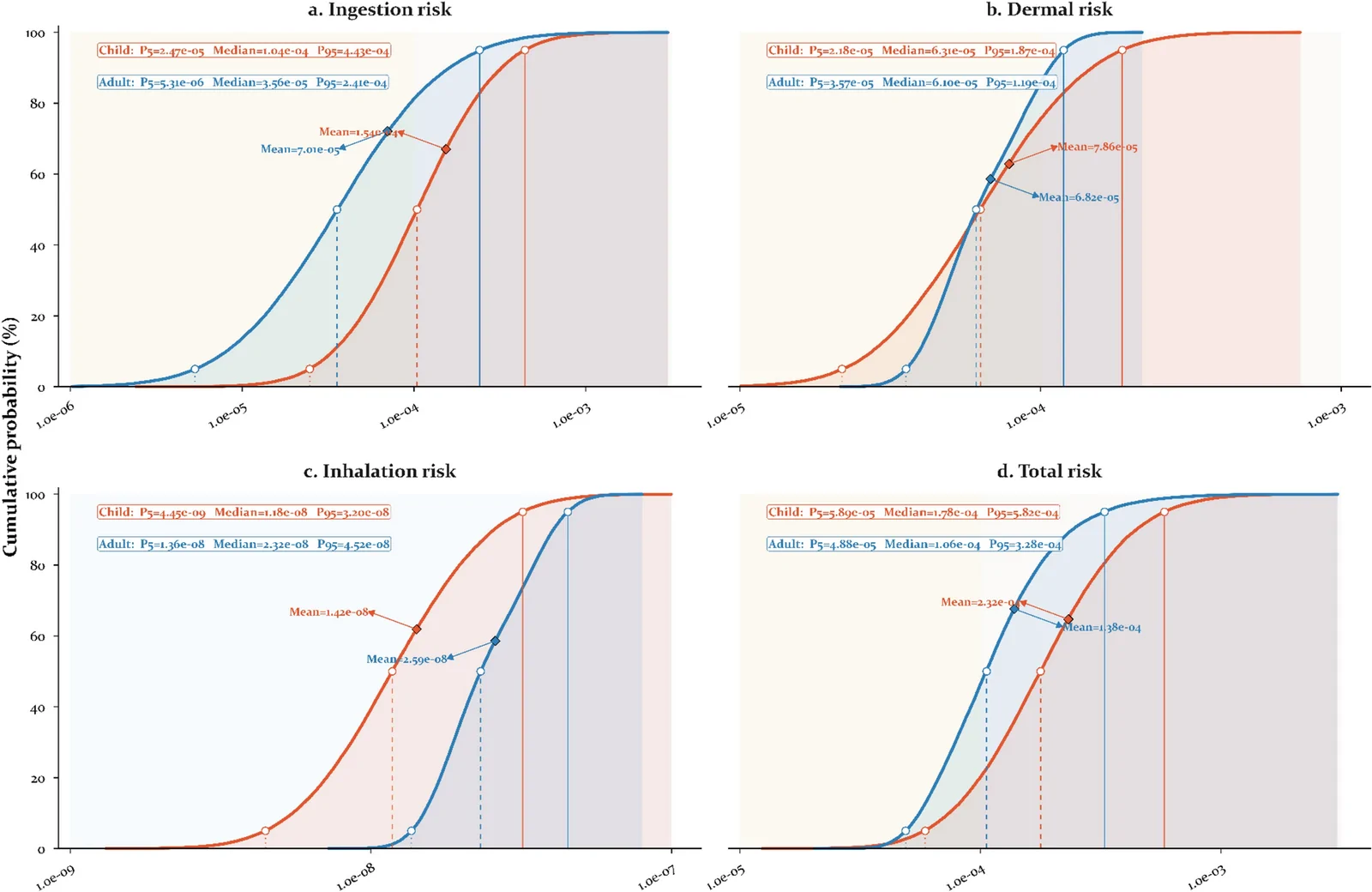
Cumulative probability distributions of CR for children and adults under the primary total As scenario: **a** CR_ing_, **b** CR_derm_, **c** CR_inh_, and **d** CR_total_. Distribution was summarized using the 5th, median, mean, and 95th percentiles. The thresholds of 10^−6^ and 10^−4^ indicate the applied CR interpretation range

The deterministically estimated CR_ing_, CR_derm_, CR_inh,_ were 3.78 × 10^–4^, 7.75 × 10^–5^ and 1.40 × 10^–8^ for children and 2.27 × 10^–4^, 6.63 × 10^–5^ and 2.52 × 10^–8^ for adults while probabilistic estimates were 4.43 × 10^–4^, 1.87 × 10^–4^, and 3.20 × 10^–8^ for children and 2.41 × 10^–4^, 1.19 × 10^–4^, and 4.52 × 10^–8^ for adults. The probabilistic P95 estimates were higher than the deterministic values for both adults (12%) and children (28%). For children, CR_total_ values were 4.56 × 10^–4^ and 5.82 × 10^–4^ for both deterministic and probabilistic estimates, respectively, while for adults, they were 2.93 × 10^–4^ and 3.28 × 10^–4^. However, the deterministic values are all above the median values and below the 95th-percentile distributions, a position that is structurally determined by right-skewed input propagation, placing fixed-input scenarios in the upper tail rather than at the central tendency of a screening assessment. This is consistent with the result of Rajasekhar et al. (2018) in an HHRA study using MCS. Considering that a CR at or above 10⁻^4^ is conventionally treated as unacceptable, the probabilistic estimation shows that both children and adults are above the limits that may represent a potential carcinogenic risk (Fig. 3). Even the 5th-percentile cancer risk values, representing the low end of the simulated exposure distribution, exceeded the 10⁻⁶ safety threshold in both deterministic and probabilistic models. However, the 95th percentile values exceed the 10^–4^ risk management reference level, which is an indication of an increased carcinogenic risk. For children, the 95th-percentile values for ingestion, dermal contact, and inhalation were 443, 187, and 0.03 times the safety threshold, respectively. Corresponding adult values were 241, 119, and 0.05-fold above the safety threshold, respectively. These results are not dependent on specific input selections but are a direct product of the ingestion cancer slope factor acting on the lifetime average daily dose distribution. The lifetime average daily dose distribution is itself amplified by the high soil As loading in the Radość allotment complex, with a mean of 698 mgkg⁻^1^. This further underscores the dominance of the ingestion pathway in cancer risk. The percentage of simulated exposure profiles falling at or above the 10⁻^4^ cancer risk threshold provides the exceedance-frequency layer that supports the interpretation of the point estimates. Approximately 80% of the simulated child exposure profiles produced total cancer risk estimates of at least 10⁻^4^, with the remaining 20% falling within the lower risk range bounded by 10⁻^5^ and 10⁻^4^. Approximately 54% of adult profiles fell within the critical cancer risk zone, while the other 46% were at the upper end of the lower risk zone. These exceedance frequencies serve as benchmark-driven severity inputs rather than categorical binary outputs, describing how often the simulated receptor population crosses the 10⁻^4^ threshold and providing a likelihood-based foundation that a single deterministic point estimate cannot provide. This is in line with interpretations in previous studies that use exceedance frequencies as severity indicators for risk communication (Mendoza-Lagunas et al., 2025; Miletić et al., 2025; Tokatlı et al., 2026). Given that both children and adults exceed the threshold indicating a potential carcinogenic effect, there is a need to mitigate public exposure to As from contaminated soils in allotment gardens.

### Influence of IVBA adjustment on ingestion-related health risks

**Fig. 4:**
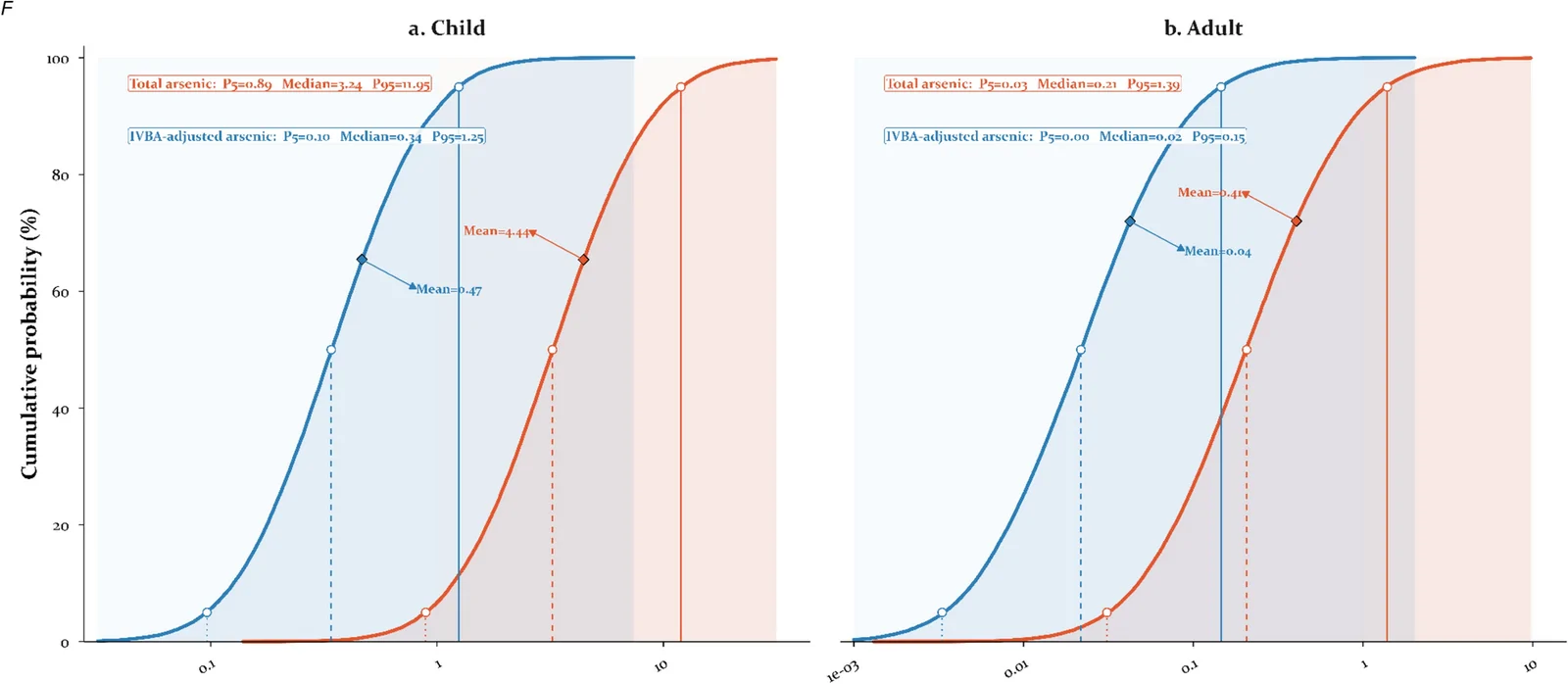
Paired probabilistic distributions of HQ_ing_ for children and adults calculated using total As and IVBA-adjusted As. The IVBA-adjusted ingestion concentration was calculated as the product of the sampled total As and its corresponding IVBA fraction. Identical soil and exposure-factor draw were retained within every paired Monte Carlo iteration; therefore, only the concentration basis used for ingestion differed between the paired estimates. Each distribution is summarized by the 5th, median, mean, and 95th percentiles. Deterministic point estimates are shown for comparison. The reference value of 1 represents the HQ screening benchmark

**Fig. 5:**
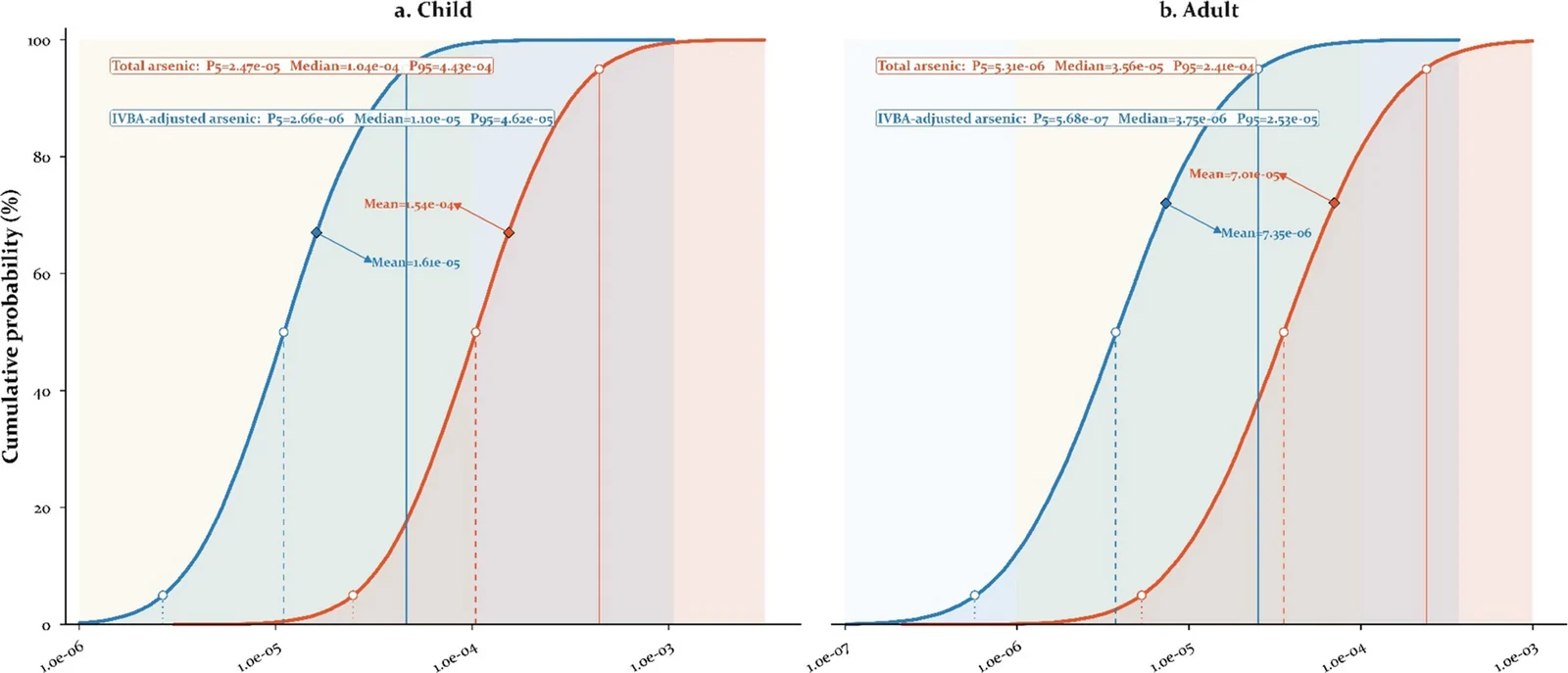
Paired probabilistic distributions of ingestion-related CR_total_ for children and adults calculated using total As and IVBA-adjusted As. Each distribution is summarized by the 5th percentile, median, mean, and 95th percentile. Deterministic point estimates are shown for comparison. Reference thresholds of 10^−6^ and 10^−4^ indicate the applied CR interpretation range

Table 5 presents results showing that applying in vitro bioaccessibility (IVBA) to the soil ingestion pathway substantially reduced the estimated As dose and associated health risks. The probabilistic median HQ_ing_ decreased from 3.24 to 0.34 for children and from 0.21 to 0.02 for adults. CR_ing_ similarly declined from 1.04 × 10^−4^ to 1.10 × 10^−5^ for children and from 3.56 × 10^−5^ to 3.75 × 10^−6^ for adults, as shown in Fig. 4 and 5. The median IVBA-adjusted to total As risk ratio was 0.11, corresponding to an 89.3% median reduction and a 9.36-fold overestimation when total As was assumed to be 100% available for ingestion. Barrio-Parra et al. (2024) noted that incorporating As bioaccessibility in playground risk assessments commonly reduced estimated risk by approximately 70–90%. It was also noted that omitting bioaccessibility in probabilistic assessments resulted in up to a 2.40-fold overestimate. Similarly, Shentu et al. (2023) reported reductions of 45.3–88.0% in children’s HI and 73.9–83.5% in children’s CR after incorporating gastrointestinal bioaccessibility into assessments of contaminated industrial soils. IVBA adjustment also changed the regulatory interpretation of the risks. For children, the percentage of simulations with ingestion HQ above 1 declined from 93.3 to 8.7%, and 84.6% of paired simulations moved from HQ > 1 to HQ ≤ 1. The proportion with cancer risk at or above 10−^4^ decreased from 51.9 to 0.6%. For adults, HQ exceedance declined from 8.7 to 0.1%, while cancer-risk exceedance decreased from 18.7 to 0.2%. Comparable changes were reported by Cocerva et al. (2024), who found that incorporating oral bioaccessibility reduced the number of urban sites identified as potentially risky by 45% and that all samples exceeding the As generic assessment criterion no longer exceeded their minimum-risk criterion after refinement. Furthermore, Różański et al. (2021) demonstrated that bioaccessible concentrations, rather than total concentrations alone, provide a more exposure-relevant basis for assessing childhood soil ingestion in Polish urban soils. However, IVBA-adjusted risks should not be interpreted as proof of the absence of risk because the median child CR remained above 10^−5^, and 53.7% of child simulations remained between 10^−5^ and 10^−4^. Thus, IVBA refinement substantially reduces estimated ingestion risk while still supporting exposure-control measures in the highly contaminated allotment soils.

**Table 5:** Ingestion-related risk calculated using IVBA-adjusted As

Receptor	Endpoint	Concentration basis	Deterministic point estimate	P05	Median	Mean	P95	Above benchmark (%)
Child	HQ	IVBA-adjusted As	1.14	0.10	0.34	0.47	1.25	8.6
Adult	HQ	IVBA-adjusted As	0.14	3.00 × 10⁻^3^	0.02	0.04	0.15	0.1
Child	CR	IVBA-adjusted As	3.95 × 10⁻^5^	2.66 × 10⁻⁶	1.10 × 10⁻^5^	1.61 × 10⁻^5^	4.62 × 10⁻^5^	0.6
Adult	CR	IVBA-adjusted As	2.37 × 10⁻^5^	5.68 × 10⁻⁷	3.75 × 10⁻⁶	7.35 × 10⁻⁶	2.53 × 10⁻^5^	0.2

The IVBA-adjusted ingestion concentration was calculated as the product of the sampled total-As concentration and its paired IVBA fraction

### Sensitivity analysis

**Fig. 6:**
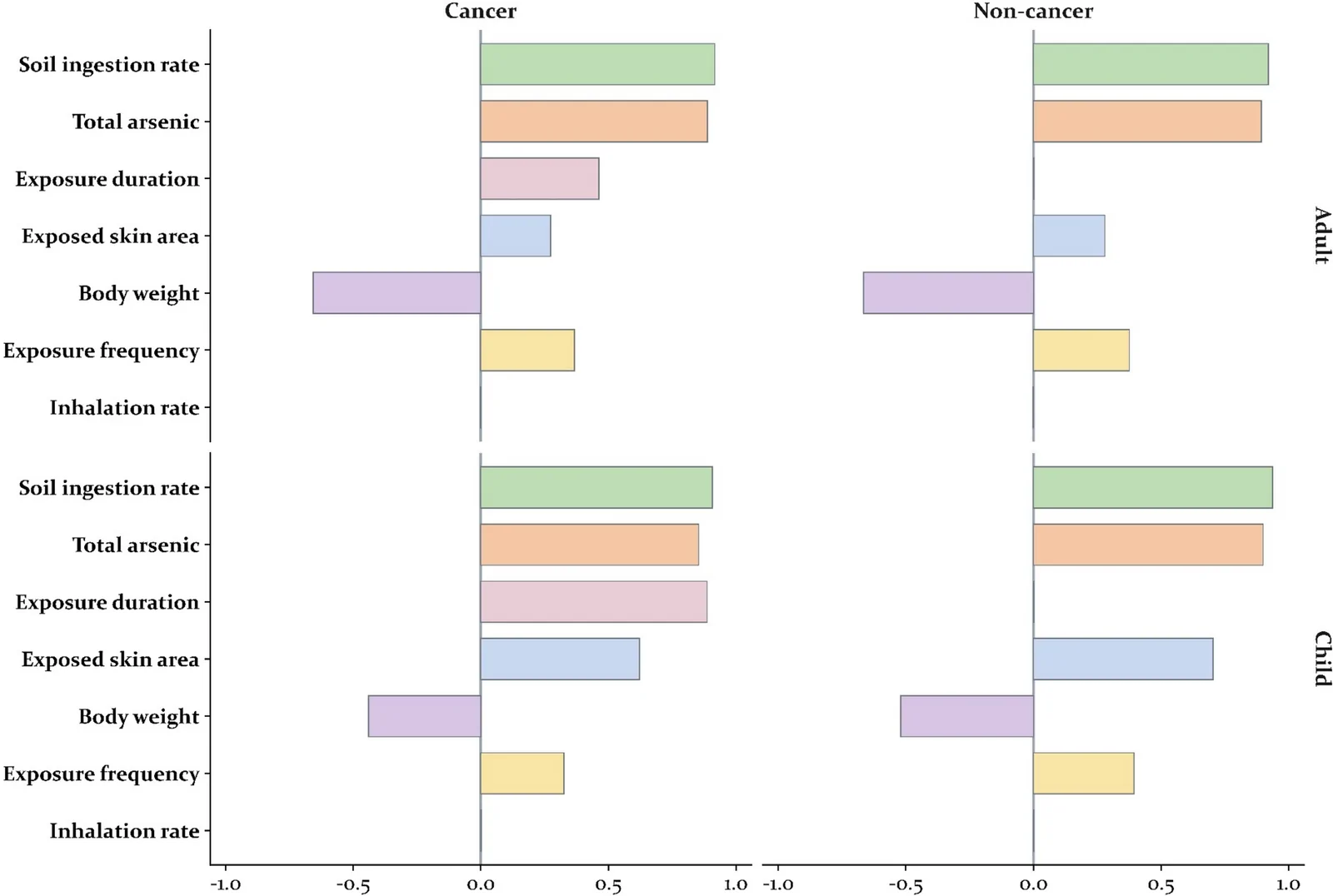
Partial rank correlation coefficients between probabilistic input variables and HI/CR_total_ risk for children and adults under the primary total As multi-pathway scenario. Positive coefficients indicate that an increase in an input was associated with an increase in modeled risk after adjustment for the remaining inputs, whereas negative coefficients indicate an inverse association. The absolute coefficient magnitude reflects the relative influence of each input within the specified model

A sensitivity analysis was conducted using partial rank correlation coefficient (PRCC) to identify the prediction parameters that contribute most to probabilistic HHRA model predictions. The results are presented as PRCC plot in Fig. 6 for both children and adults. The sensitivity analysis identified total As and soil-ingestion rate as strong positive drivers of HI and CR_total_. For children HI, the largest positive coefficients were for soil-ingestion rate (0.94), total As (0.90) and exposed skin area (0.70), while body weight was inversely associated (− 0.52). For children CR, soil-ingestion rate (0.91), exposure duration (0.89), and total As (0.85) were dominant. Adult results showed the same pattern as in soil ingestion rate and total As: PRCCs of approximately 0.89–0.92, with body weight showing a strong negative effect. The inhalation rate was close to zero for all total-risk endpoints. This indicates that the risk of cancer increases as the soil As concentration rises. Therefore, health risk mitigation strategies and plans should focus more on reducing concentrations in soil.

### Remedial goal

As it has been established that a considerable percentage of the population is at risk of cancer due to exposure to As-contaminated soil in the allotment garden complex, a remedial goal (RG) for this site was estimated. To derive and identify soil concentrations that would limit exposure to acceptable levels. This form of preliminary management and remediation estimates provides the first line of action towards mitigating the adverse human health impacts of As exposure. According to the Polish soil quality benchmark, permissible As concentrations are set at 20 mgkg^−1^ for soils used for both agricultural and horticultural purposes, as well as for soils in recreational areas (Rozporządzenie Ministra Środowiska, 2002). As presented in Table 6, at a target cancer risk of 10^−5^, the deterministic total As multi-pathway RGs were 15.31 mgkg^−1^ for children and 23.80 mgkg^−1^ for adults. The probabilistic 5th percentile (P05) values were 12.99 mgkg^−1^ for children and 22.75 mgkg^−1^ for adults, while the corresponding medians were 37.40 and 65.22 mgkg^−1^. Thus, the Polish value of 20 mgkg^−1^ lies above the conservative child deterministic and P05 estimates but below the corresponding adult values. Under the unadjusted total As model, 20 mgkg^−1^ would therefore be broadly consistent with an intermediate 10^−5^ management level, although it would not provide the same modeled protection as the child P05 value of approximately 13 mgkg^−1^. Application of IVBA to the ingestion pathway increased the multi-pathway RGs because only a fraction of total soil As was assumed to be potentially available for gastrointestinal absorption. At the 10^−5^ target, the deterministic IVBA-adjusted RGs were 59.62 mgkg^−1^ for children and 77.53 mgkg^−1^ for adults. The probabilistic P05 values were 34.80 mgkg^−1^ for children and 64.13 mgkg^−1^ for adults, with medians of 85.05 and 96.10 mgkg^−1^, respectively. Consequently, the Polish soil quality benchmark of 20 mgkg^−1^ is below both child and adult IVBA-adjusted P05 values. In probabilistic terms, a concentration of 20 mgkg^−1^ would therefore satisfy the 10^−5^ target for at least approximately 95% of the simulated exposure profiles under the IVBA-adjusted multi-pathway model. The difference between the total As child P05 RG of 12.99 mgkg^−1^ and the IVBA-adjusted value of 34.80 mgkg^−1^ illustrates the conservatism introduced when total As is assumed to be completely available through ingestion. Similar effects were reported by Yang et al. (2025) where As RG was 34.4 mgkg^−1^ using locally adapted exposure assumptions and 54 mgkg^−1^ after incorporating bioaccessibility, while regional goals ranged from 15.1 to 31.7 mgkg^−1^. The Polish soil quality benchmark of 20 mgkg^−1^ therefore lies at the lower end of the range reported for other locally derived As RG. The Polish value of 20 mgkg⁻^1^ is retained solely as an external regulatory comparison benchmark. The present results do not establish it as a definitive site-specific cleanup concentration because food-chain transfer, water exposure, spatial heterogeneity, background exposure, and remediation feasibility were not included. This value is less conservative than 13 mgkg^−1^ total As P05 estimate, and it is consistent with the Polish soil quality benchmark. However, it remains below the IVBA-adjusted child P05 RG of 34.80 mg mgkg^−1^. It also represents a compromise between conservative total-concentration screening and site-specific bioaccessibility refinement. It should be interpreted as an operational management target associated with the approximately 10^−5^ risk level, rather than the much stricter 10^−6^ level. At 10^−6^, even the IVBA-adjusted P05 RGs were only 3.48 mgkg^−1^ for children and 6.41 mgkg^−1^ for adults, indicating that 20 mgkg^−1^ would not meet the highly conservative target. This result supports active exposure management, including targeted removal of highly contaminated soil, clean-soil replacement or capping, raised cultivation beds, dust suppression, and reduced direct soil contact by children. The proposed 20 mgkg^−1^ RG should nevertheless remain a screening and management value rather than an automatically enforceable cleanup endpoint.

**Table 6:** Deterministic point estimates and probabilistic distributions of total multi-pathway remedial goals for children and adults at target lifetime cancer risks of 10^−6^, 10^−5^, and 10^−4^

Model	Receptor	Target cancer risk	Deterministic	P05	Median	Mean	P95
Total-As multi-pathway	Child	10⁻⁶	1.53	1.3	3.74	4.44	9.92
Total-As multi-pathway	Child	10⁻^5^	15.31	12.99	37.40	44.37	99.17
Total-As multi-pathway	Child	10⁻^4^	153	130	374	444	992
Total-As multi-pathway	Adult	10⁻⁶	2.38	2.27	6.52	6.57	11.10
Total-As multi-pathway	Adult	10⁻^5^	23.80	22.75	65.22	65.70	111
Total-As multi-pathway	Adult	10⁻^4^	238	228	652	657	1110
IVBA-adjusted ingestion multi-pathway	Child	10⁻⁶	5.96	3.48	8.51	9.86	20.80
IVBA-adjusted ingestion multi-pathway	Child	10⁻^5^	59.62	34.80	85.05	98.59	208
IVBA-adjusted ingestion multi-pathway	Child	10⁻^4^	596	348	851	986	2080
IVBA-adjusted ingestion multi-pathway	Adult	10⁻⁶	7.75	6.41	9.61	9.78	13.73
IVBA-adjusted ingestion multi-pathway	Adult	10⁻^5^	77.53	64.13	96.10	97.76	137
IVBA-adjusted ingestion multi-pathway	Adult	10⁻^4^	775	641	961	978	1373

Probabilistic remedial goals were calculated from the corresponding total cancer-risk coefficient and were subsequently summarized by the 5th percentile, median, arithmetic mean, and 95th percentile. Lower remedial goal values are more health-protective because they correspond to larger exposure and risk coefficients; consequently, the 5th-percentile remedial goal represents the protective lower-tail concentration. In the IVBA-refined model, IVBA was applied only to the ingestion coefficient, while dermal contact and inhalation remained based on total As. All values are expressed as mgkg^−1^

### Study limitations

While this study provides valuable insights into HHRA in contaminated garden soils, the importance of incorporating IVBA into risk assessments and using probabilistic estimates should be recognized, given several remaining limitations. Although the sample size in this study captured substantial variation in total As and IVBA, it may not fully represent the spatial heterogeneity of the allotments or the wider Złoty Stok mining area. The samples were treated as exchangeable observations from a pooled site-wide distribution, without modeling plot-level differences, spatial autocorrelation, or the sizes of contaminated hotspots. Sampling was also insufficient to quantify seasonal changes associated with soil moisture, redox conditions, gardening activity, or weather. Geochemical interpretation is also constrained by the available measurements. Total As cannot be used to distinguish between As oxidation states, mineral forms, or binding environments, which can differ considerably in mobility, toxicity, and gastric solubility. Information regarding solid-phase speciation and mineralogical characterization was unavailable. Therefore, the retention of As in less-mobile solid-phase minerals remains a plausible explanation for the relatively low IVBA, rather than a directly confirmed sample-specific mechanism. Quantitative receptor modeling may further strengthen probabilistic assessments by estimating the contributions of different contamination sources and ecotoxicological indices to the resulting health risks (Tokatlı et al., 2026; Ustaoğlu et al., 2026). The exposure assessment included direct soil ingestion, dermal contact, and inhalation, but did not incorporate consumption of vegetables grown in the allotments. Exposure through water sources was also excluded. Furthermore, the assessment did not evaluate co-exposure to other contaminants associated with historical mining. Therefore, future research should address these limitations.

## Conclusion

This study demonstrated that soils from the Radość allotment-garden complex are highly enriched in As and that exposure to these soils may require risk-management attention, particularly for children. Under the total-As multi-pathway model, the median HI exceeded 1 for children but remained below 1 for adults, although the upper tail of the adult distribution also exceeded this threshold. Median CR_total_ estimates were above 10⁻^4^ for both receptor groups. The deterministic results generally represented upper-tail exposure scenarios, whereas MCS characterized the broader distribution of possible outcomes and identified the inputs that most strongly influenced the estimates. The probabilistic approach should therefore be regarded as more informative for representing variability and supporting sensitivity analysis. Total soil As concentration and soil-ingestion rate were the principal positive risk drivers; body weight was inversely associated with risk, and inhalation contributed negligibly under the adopted assumptions. Ingestion and dermal contact together accounted for nearly all modeled multi-pathway risk. Applying the measured IVBA fraction to ingestion reduced the median ingestion-related HQ and CR risk by 89.3%. This result demonstrates the importance of considering contaminant solubilization under simulated gastric conditions when refining soil-ingestion assessments. At a target CR of 10⁻^5^, the child P05 remedial goal was approximately 13.0 mgkg⁻^1^ under the total-As multi-pathway model and 34.8 mgkg⁻^1^ when IVBA was applied only to ingestion. These values illustrate RG’s sensitivity to the concentration basis, exposure assumptions, and the selected target risk. Therefore, the Polish soil-quality value of 20 mgkg⁻^1^ should be retained only as an external regulatory comparison. These RG estimates should be considered preliminary direct-soil-exposure screening values, as final remediation decisions require additional spatial sampling, verification of mineralogical controls, locally measured exposure behavior, and assessment of vegetable, water, and multi-element exposure.

## Supplementary Information

Below is the link to the electronic supplementary material.

Supplementary file1 (DOCX 103 KB)

## Data Availability

The data used in the analysis are provided in the Supplementary Information.
