# Supplementary material for: Human health risk and remedial goal assessment using Monte Carlo simulation: a case study of arsenic contaminated allotment gardens in southern Poland
Source: Environ Geochem Health. 2026 Sep 4;48(14):571. doi: 10.1007/s10653-026-03462-8 (PMC13545054; doi:10.1007/s10653-026-03462-8)
Supplement: Supplementary file 1 [file 10653_2026_3462_MOESM1_ESM.docx]

Title:

Human health risk and remedial goal assessment using Monte Carlo Simulation: A case study of arsenic contaminated allotment gardens in southern Poland

Authors:

Kayode Olabode^1*^, Karolina Lewińska^1^, Izabela Komorowicz^2^

*Corresponding author - [kayola@amu.edu.pl](mailto:kayola@amu.edu.pl)

Affiliations:

^1^Faculty of Geographical and Geological Sciences, Adam Mickiewicz University, Krygowskiego 12, 61-680 Poznań, Poland

^2^Faculty of Chemistry, Adam Mickiewicz University, Uniwersytetu Poznańskiego 8, 61-614 Poznań, Poland

ORCID:

Kayode Olabode: 0009-0007-5139-8185

Karolina Lewińska: 0000-0002-9630-4855

Izabela Komorowicz: 0000-0002-0037-5908

**Supplementary information**

Table S1. List of abbreviations.

| **Abbreviation** | **Definition** |
| --- | --- |
| ABSd | Dermal absorption fraction |
| ADD | Average daily dose |
| AF | Soil-to-skin adherence factor |
| As | Arsenic |
| AT | Averaging time |
| ATca | Cancer averaging time |
| ATnc | Non-cancer averaging time |
| BW | Body weight |
| CF | Unit conversion factor |
| CI | Confidence interval |
| CMRG | Combined multiple recursive generator |
| CR | Cancer risk |
| CR_total_ | Total cancer risk |
| CRM | Certified reference material |
| CV | Coefficient of variation |
| ED | Exposure duration |
| EF | Exposure frequency |
| HHRA | Human health risk assessment |
| HI | Hazard index |
| HQ | Hazard quotient |
| ICP-MS | Inductively coupled plasma mass spectrometry |
| iCRC | Integrated collision reaction cell |
| IR_ing_ | Soil ingestion rate |
| IR_inh_ | Inhalation rate |
| IR_soil_ | Soil ingestion rate used in the supplementary tables |
| IVBA | In vitro bioaccessibility |
| LADD | Lifetime average daily dose |
| LOD | Limit of detection |
| LOQ | Limit of quantification |
| MCS | Monte Carlo simulation |
| NIST | National Institute of Standards and Technology |
| P05 | 5th percentile |
| P95 | 95th percentile |
| PEF | Particulate emission factor |
| PRCC | Partial rank correlation coefficient |
| QA/QC | Quality assurance and quality control |
| R | Pearson correlation coefficient |
| R² | Coefficient of determination |
| RfD | Reference dose |
| RG | Remedial goal |
| SA | Exposed skin area |
| SBET | Simplified Bioaccessibility Extraction Test |
| SD | Standard deviation |
| SF | Cancer slope factor |
| SRM | Standard reference material |
| TR | Target lifetime cancer risk |
| W | Shapiro-Wilk test statistic |
| ρ | Spearman rank correlation coefficient |

Table S2. Configuration of the joint paired smoothed empirical resampling model. Total As and in vitro bioaccessibility (IVBA) were resampled jointly to preserve their measured dependence.

| **Setting** | **Value** |
| --- | --- |
| Assessment scale | Pooled site-wide; no plot-level or spatial inference |
| As concentrations sampling model | Joint paired smoothed empirical bootstrap |
| Transformation for total arsenic | Natural logarithm |
| Transformation for IVBA | Logit |
| Scott bandwidth factor | 0.550321 |
| Kernel multiplier | 0.500 |
| Effective smoothing factor | 0.275161 |
| Boundary handling | Reflection within the measured transformed ranges |
| Dependence preservation | Correlated bivariate kernel perturbation |

Table S3. Comparison of measured and simulated environmental input distributions. Total As and bioaccessible As are expressed in mg kg−1; IVBA is expressed as a fraction.

| **Variable** | **Measured mean** | **Simulated mean** | **Measured P05** | **Simulated P05** | **Measured P95** | **Simulated P95** |
| --- | --- | --- | --- | --- | --- | --- |
| Total As | 698.08333 | 701.48516 | 435.9 | 443.56646 | 1056.7 | 1093.35112 |
| IVBA | 0.11106 | 0.11101 | 0.05535 | 0.05645 | 0.20624 | 0.19822 |
| Bioaccessible As | 72.93917 | 73.53263 | 50.367 | 48.39044 | 115.25 | 117.60851 |

Table S4. Dependence between total As and IVBA in the measured and simulated datasets. Pearson correlations were calculated using log-transformed total As and logit-transformed IVBA. Spearman correlations were calculated on the original scales.

| **Dataset** | **Pearson r (log total As, logit IVBA)** | **Spearman ρ (total As, IVBA)** |
| --- | --- | --- |
| Measured pooled data | -0.5994 | -0.6353 |
| Smoothed pooled simulation | -0.5723 | -0.5902 |

Table S5. Shapiro–Wilk tests for total As, bioaccessible As and IVBA. A p-value below 0.05 indicates rejection of the null hypothesis of normality.

| **Variable** | **Scale** | **Shapiro–Wilk W** | **p-value** | **Interpretation** |
| --- | --- | --- | --- | --- |
| Total As | Raw | 0.8471 | 1.63 × 10⁻⁴ | Normality rejected |
| Total As | Natural log | 0.8677 | 5 × 10⁻⁴ | Normality rejected |
| Bioaccessible As | Raw | 0.846 | 1.54 × 10⁻⁴ | Normality rejected |
| Bioaccessible As | Natural log | 0.8862 | 0.0015 | Normality rejected |
| IVBA | Raw fraction | 0.8734 | 6.92 × 10⁻⁴ | Normality rejected |
| IVBA | Logit | 0.9416 | 0.0571 | Normality not rejected |

Table S6. Correlation between total As concentration and IVBA. Pearson "r" is reported with its 95% confidence interval. The Spearman coefficient is based on ranked observations.

| **Method** | **Coefficient** | **95% CI lower** | **95% CI upper** | **p-value** |
| --- | --- | --- | --- | --- |
| Pearson | -0.536 | -0.735 | -0.252 | 7.51 × 10⁻⁴ |
| Spearman | -0.635 | — | — | 3.15 × 10⁻⁵ |

Table S7. Target, deterministic, and simulated values of receptor exposure variables. Target values define the assigned probability distributions.

| **Receptor** | **Variable** | **Unit** | **Target mean** | **Target P95** | **Deterministic** | **Simulated mean** | **Simulated SD** | **Simulated P05** | **Simulated median** | **Simulated P95** |
| --- | --- | --- | --- | --- | --- | --- | --- | --- | --- | --- |
| Child | Soil ingestion rate | mg day⁻¹ | 80 | 200 | 200 | 80.0787 | 64.7029 | 19.3218 | 62.1693 | 200.1311 |
| Adult | Soil ingestion rate | mg day⁻¹ | 30 | 100 | 100 | 30.0577 | 46.3547 | 2.6974 | 16.3518 | 100.21 |
| Child | Body weight | kg | 16.8 | — | 16.8 | 16.7956 | 1.6755 | 14.1831 | 16.7151 | 19.6836 |
| Adult | Body weight | kg | 70 | — | 70 | 69.9972 | 10.7092 | 53.8396 | 69.1829 | 88.8932 |
| Child | Exposed skin area | cm² | 2800 | — | 2800 | 2801.8089 | 1178.8451 | 1334.4079 | 2583.5647 | 5020.9101 |
| Adult | Exposed skin area | cm² | 5700 | — | 5700 | 5701.6945 | 440.4436 | 5010.1698 | 5684.2739 | 6455.7661 |
| Child | Inhalation rate | m³ day⁻¹ | 10 | — | 10 | 9.996 | 3.1528 | 5.7509 | 9.5313 | 15.8428 |
| Adult | Inhalation rate | m³ day⁻¹ | 15 | — | 15 | 14.9996 | 0.9544 | 13.4818 | 14.9664 | 16.6255 |
| Child | Exposure duration | years | 6 | — | 6 | 5.9919 | 2.3804 | 2.9578 | 5.5741 | 10.446 |
| Adult | Exposure duration | years | 30 | — | 30 | 29.9929 | 2.7331 | 25.7215 | 29.8716 | 34.6796 |
| Both | Exposure frequency | days year⁻¹ | 144 | — | 144 | 143.9537 | 9.7868 | 127.5726 | 143.9476 | 160.3753 |

Table S8. Environmental and exposure inputs used in the deterministic total-As and IVBA-adjusted risk scenarios. The IVBA adjustment was applied only to ingestion concentration; dermal contact and inhalation calculations retained total As.

| **Scenario** | **Receptor** | **Total As** | **Bioaccessible As** | **Mean measured IVBA** | **Effective IVBA** | **Ingestion As** | **IRsoil** | **EF** | **BW** | **SA** | **AF** | **IRinh** | **ED** |
| --- | --- | --- | --- | --- | --- | --- | --- | --- | --- | --- | --- | --- | --- |
| IVBA-adjusted ingestion; total As for dermal/inhalation | Child | 698.08 | 72.94 | 0.1111 | 0.1045 | 72.94 | 200 | 144 | 16.8 | 2800 | 0.2 | 10 | 6 |
| IVBA-adjusted ingestion; total As for dermal/inhalation | Adult | 698.08 | 72.94 | 0.1111 | 0.1045 | 72.94 | 100 | 144 | 70 | 5700 | 0.07 | 15 | 30 |
| Total-As multi-pathway | Child | 698.08 | 72.94 | 0.1111 | 0.1045 | 698.08 | 200 | 144 | 16.8 | 2800 | 0.2 | 10 | 6 |
| Total-As multi-pathway | Adult | 698.08 | 72.94 | 0.1111 | 0.1045 | 698.08 | 100 | 144 | 70 | 5700 | 0.07 | 15 | 30 |

Table S9. Convergence of total hazard index and total cancer risk with increasing Monte Carlo iterations. Convergence was evaluated using the mean, median, and P95 at iteration checkpoints from 1,000 to 200,000.

| **Receptor** | **Endpoint** | **Iterations** | **Mean** | **Median** | **P95** |
| --- | --- | --- | --- | --- | --- |
| Child | HI | 1,000 | 6.39 | 5.471 | 14.523 |
| Child | HI | 5,000 | 6.649 | 5.435 | 15.139 |
| Child | HI | 10,000 | 6.693 | 5.493 | 15.217 |
| Child | HI | 25,000 | 6.67 | 5.488 | 15.155 |
| Child | HI | 50,000 | 6.686 | 5.497 | 15.136 |
| Child | HI | 100,000 | 6.707 | 5.493 | 15.178 |
| Child | HI | 200,000 | 6.715 | 5.503 | 15.178 |
| Child | CR_total_ | 1,000 | 2.19 × 10⁻⁴ | 1.77 × 10⁻⁴ | 5.41 × 10⁻⁴ |
| Child | CR_total_ | 5,000 | 2.28 × 10⁻⁴ | 1.76 × 10⁻⁴ | 5.67 × 10⁻⁴ |
| Child | CR_total_ | 10,000 | 2.31 × 10⁻⁴ | 1.77 × 10⁻⁴ | 5.76 × 10⁻⁴ |
| Child | CR_total_ | 25,000 | 2.31 × 10⁻⁴ | 1.77 × 10⁻⁴ | 5.85 × 10⁻⁴ |
| Child | CR_total_ | 50,000 | 2.32 × 10⁻⁴ | 1.77 × 10⁻⁴ | 5.86 × 10⁻⁴ |
| Child | CR_total_ | 100,000 | 2.32 × 10⁻⁴ | 1.77 × 10⁻⁴ | 5.86 × 10⁻⁴ |
| Child | CR_total_ | 200,000 | 2.32 × 10⁻⁴ | 1.78 × 10⁻⁴ | 5.82 × 10⁻⁴ |
| Adult | HI | 1,000 | 0.809 | 0.617 | 1.911 |
| Adult | HI | 5,000 | 0.799 | 0.619 | 1.893 |
| Adult | HI | 10,000 | 0.79 | 0.616 | 1.862 |
| Adult | HI | 25,000 | 0.79 | 0.613 | 1.847 |
| Adult | HI | 50,000 | 0.799 | 0.614 | 1.879 |
| Adult | HI | 100,000 | 0.799 | 0.615 | 1.884 |
| Adult | HI | 200,000 | 0.799 | 0.616 | 1.889 |
| Adult | CR_total_ | 1,000 | 1.4 × 10⁻⁴ | 1.06 × 10⁻⁴ | 3.31 × 10⁻⁴ |
| Adult | CR_total_ | 5,000 | 1.38 × 10⁻⁴ | 1.07 × 10⁻⁴ | 3.29 × 10⁻⁴ |
| Adult | CR_total_ | 10,000 | 1.37 × 10⁻⁴ | 1.06 × 10⁻⁴ | 3.27 × 10⁻⁴ |
| Adult | CR_total_ | 25,000 | 1.37 × 10⁻⁴ | 1.05 × 10⁻⁴ | 3.22 × 10⁻⁴ |
| Adult | CR_total_ | 50,000 | 1.38 × 10⁻⁴ | 1.06 × 10⁻⁴ | 3.27 × 10⁻⁴ |
| Adult | CR_total_ | 100,000 | 1.38 × 10⁻⁴ | 1.06 × 10⁻⁴ | 3.28 × 10⁻⁴ |
| Adult | CR_total_ | 200,000 | 1.38 × 10⁻⁴ | 1.06 × 10⁻⁴ | 3.28 × 10⁻⁴ |

Table S10. Distribution of simulated lifetime cancer-risk estimates across predefined risk intervals for children and adults under total-As and IVBA-adjusted exposure scenarios. Percentages represent the empirical frequency of Monte Carlo iterations falling within four mutually exclusive lifetime cancer-risk intervals (CR < 10^−6^, 10^−6^ ≤ CR < 10^−5^, 10^−5^ ≤ CR< 10^−4^, and CR ≥ 10^−4^). Results are reported separately for ingestion, dermal contact, inhalation, and multi-pathway. Percentages within each receptor, scenario, and pathway sum to approximately 100%.

| **Analysis** | **Scenario** | **Receptor** | **Endpoint** | **Pathway interval** | **Percentage** |
| --- | --- | --- | --- | --- | --- |
| Cancer-risk interval frequency | Total-As multi-pathway | Child | Cancer | Ingestion: < 10⁻⁶ | 0.0 |
| Cancer-risk interval frequency | Total-As multi-pathway | Child | Cancer | Ingestion: 10⁻⁶ to < 10⁻⁵ | 0.4 |
| Cancer-risk interval frequency | Total-As multi-pathway | Child | Cancer | Ingestion: 10⁻⁵ to < 10⁻⁴ | 47.8 |
| Cancer-risk interval frequency | Total-As multi-pathway | Child | Cancer | Ingestion: ≥ 10⁻⁴ | 51.9 |
| Cancer-risk interval frequency | Total-As multi-pathway | Child | Cancer | Dermal contact: < 10⁻⁶ | 0.0 |
| Cancer-risk interval frequency | Total-As multi-pathway | Child | Cancer | Dermal contact: 10⁻⁶ to < 10⁻⁵ | 0.2 |
| Cancer-risk interval frequency | Total-As multi-pathway | Child | Cancer | Dermal contact: 10⁻⁵ to < 10⁻⁴ | 75.3 |
| Cancer-risk interval frequency | Total-As multi-pathway | Child | Cancer | Dermal contact: ≥ 10⁻⁴ | 24.5 |
| Cancer-risk interval frequency | Total-As multi-pathway | Child | Cancer | Inhalation: < 10⁻⁶ | 100.0 |
| Cancer-risk interval frequency | Total-As multi-pathway | Child | Cancer | Inhalation: 10⁻⁶ to < 10⁻⁵ | 0.0 |
| Cancer-risk interval frequency | Total-As multi-pathway | Child | Cancer | Inhalation: 10⁻⁵ to < 10⁻⁴ | 0.0 |
| Cancer-risk interval frequency | Total-As multi-pathway | Child | Cancer | Inhalation: ≥ 10⁻⁴ | 0.0 |
| Cancer-risk interval frequency | Total-As multi-pathway | Child | Cancer | Multi-pathway: < 10⁻⁶ | 0.0 |
| Cancer-risk interval frequency | Total-As multi-pathway | Child | Cancer | Multi-pathway: 10⁻⁶ to < 10⁻⁵ | 0.0 |
| Cancer-risk interval frequency | Total-As multi-pathway | Child | Cancer | Multi-pathway: 10⁻⁵ to < 10⁻⁴ | 20.0 |
| Cancer-risk interval frequency | Total-As multi-pathway | Child | Cancer | Multi-pathway: ≥ 10⁻⁴ | 80.0 |
| Cancer-risk interval frequency | Total-As multi-pathway | Adult | Cancer | Ingestion: < 10⁻⁶ | 0.1 |
| Cancer-risk interval frequency | Total-As multi-pathway | Adult | Cancer | Ingestion: 10⁻⁶ to < 10⁻⁵ | 13.6 |
| Cancer-risk interval frequency | Total-As multi-pathway | Adult | Cancer | Ingestion: 10⁻⁵ to < 10⁻⁴ | 67.6 |
| Cancer-risk interval frequency | Total-As multi-pathway | Adult | Cancer | Ingestion: ≥ 10⁻⁴ | 18.7 |
| Cancer-risk interval frequency | Total-As multi-pathway | Adult | Cancer | Dermal contact: < 10⁻⁶ | 0.0 |
| Cancer-risk interval frequency | Total-As multi-pathway | Adult | Cancer | Dermal contact: 10⁻⁶ to < 10⁻⁵ | 0.0 |
| Cancer-risk interval frequency | Total-As multi-pathway | Adult | Cancer | Dermal contact: 10⁻⁵ to < 10⁻⁴ | 85.9 |
| Cancer-risk interval frequency | Total-As multi-pathway | Adult | Cancer | Dermal contact: ≥ 10⁻⁴ | 14.1 |
| Cancer-risk interval frequency | Total-As multi-pathway | Adult | Cancer | Inhalation: < 10⁻⁶ | 100.0 |
| Cancer-risk interval frequency | Total-As multi-pathway | Adult | Cancer | Inhalation: 10⁻⁶ to < 10⁻⁵ | 0.0 |
| Cancer-risk interval frequency | Total-As multi-pathway | Adult | Cancer | Inhalation: 10⁻⁵ to < 10⁻⁴ | 0.0 |
| Cancer-risk interval frequency | Total-As multi-pathway | Adult | Cancer | Inhalation: ≥ 10⁻⁴ | 0.0 |
| Cancer-risk interval frequency | Total-As multi-pathway | Adult | Cancer | Multi-pathway: < 10⁻⁶ | 0.0 |
| Cancer-risk interval frequency | Total-As multi-pathway | Adult | Cancer | Multi-pathway: 10⁻⁶ to < 10⁻⁵ | 0.0 |
| Cancer-risk interval frequency | Total-As multi-pathway | Adult | Cancer | Multi-pathway: 10⁻⁵ to < 10⁻⁴ | 46.0 |
| Cancer-risk interval frequency | Total-As multi-pathway | Adult | Cancer | Multi-pathway: ≥ 10⁻⁴ | 54.0 |
| Cancer-risk interval frequency | IVBA-adjusted ingestion; total As for dermal/inhalation | Child | Cancer | Ingestion: < 10⁻⁶ | 0.3 |
| Cancer-risk interval frequency | IVBA-adjusted ingestion; total As for dermal/inhalation | Child | Cancer | Ingestion: 10⁻⁶ to < 10⁻⁵ | 45.4 |
| Cancer-risk interval frequency | IVBA-adjusted ingestion; total As for dermal/inhalation | Child | Cancer | Ingestion: 10⁻⁵ to < 10⁻⁴ | 53.7 |
| Cancer-risk interval frequency | IVBA-adjusted ingestion; total As for dermal/inhalation | Child | Cancer | Ingestion: ≥ 10⁻⁴ | 0.6 |
| Cancer-risk interval frequency | IVBA-adjusted ingestion; total As for dermal/inhalation | Child | Cancer | Dermal contact: < 10⁻⁶ | 0.0 |
| Cancer-risk interval frequency | IVBA-adjusted ingestion; total As for dermal/inhalation | Child | Cancer | Dermal contact: 10⁻⁶ to < 10⁻⁵ | 0.2 |
| Cancer-risk interval frequency | IVBA-adjusted ingestion; total As for dermal/inhalation | Child | Cancer | Dermal contact: 10⁻⁵ to < 10⁻⁴ | 75.3 |
| Cancer-risk interval frequency | IVBA-adjusted ingestion; total As for dermal/inhalation | Child | Cancer | Dermal contact: ≥ 10⁻⁴ | 24.5 |
| Cancer-risk interval frequency | IVBA-adjusted ingestion; total As for dermal/inhalation | Child | Cancer | Inhalation: < 10⁻⁶ | 100.0 |
| Cancer-risk interval frequency | IVBA-adjusted ingestion; total As for dermal/inhalation | Child | Cancer | Inhalation: 10⁻⁶ to < 10⁻⁵ | 0.0 |
| Cancer-risk interval frequency | IVBA-adjusted ingestion; total As for dermal/inhalation | Child | Cancer | Inhalation: 10⁻⁵ to < 10⁻⁴ | 0.0 |
| Cancer-risk interval frequency | IVBA-adjusted ingestion; total As for dermal/inhalation | Child | Cancer | Inhalation: ≥ 10⁻⁴ | 0.0 |
| Cancer-risk interval frequency | IVBA-adjusted ingestion; total As for dermal/inhalation | Child | Cancer | Multi-pathway: < 10⁻⁶ | 0.0 |
| Cancer-risk interval frequency | IVBA-adjusted ingestion; total As for dermal/inhalation | Child | Cancer | Multi-pathway: 10⁻⁶ to < 10⁻⁵ | 0.0 |
| Cancer-risk interval frequency | IVBA-adjusted ingestion; total As for dermal/inhalation | Child | Cancer | Multi-pathway: 10⁻⁵ to < 10⁻⁴ | 65.5 |
| Cancer-risk interval frequency | IVBA-adjusted ingestion; total As for dermal/inhalation | Child | Cancer | Multi-pathway: ≥ 10⁻⁴ | 34.4 |
| Cancer-risk interval frequency | IVBA-adjusted ingestion; total As for dermal/inhalation | Adult | Cancer | Ingestion: < 10⁻⁶ | 12.5 |
| Cancer-risk interval frequency | IVBA-adjusted ingestion; total As for dermal/inhalation | Adult | Cancer | Ingestion: 10⁻⁶ to < 10⁻⁵ | 67.7 |
| Cancer-risk interval frequency | IVBA-adjusted ingestion; total As for dermal/inhalation | Adult | Cancer | Ingestion: 10⁻⁵ to < 10⁻⁴ | 19.5 |
| Cancer-risk interval frequency | IVBA-adjusted ingestion; total As for dermal/inhalation | Adult | Cancer | Ingestion: ≥ 10⁻⁴ | 0.2 |
| Cancer-risk interval frequency | IVBA-adjusted ingestion; total As for dermal/inhalation | Adult | Cancer | Dermal contact: < 10⁻⁶ | 0.0 |
| Cancer-risk interval frequency | IVBA-adjusted ingestion; total As for dermal/inhalation | Adult | Cancer | Dermal contact: 10⁻⁶ to < 10⁻⁵ | 0.0 |
| Cancer-risk interval frequency | IVBA-adjusted ingestion; total As for dermal/inhalation | Adult | Cancer | Dermal contact: 10⁻⁵ to < 10⁻⁴ | 85.9 |
| Cancer-risk interval frequency | IVBA-adjusted ingestion; total As for dermal/inhalation | Adult | Cancer | Dermal contact: ≥ 10⁻⁴ | 14.1 |
| Cancer-risk interval frequency | IVBA-adjusted ingestion; total As for dermal/inhalation | Adult | Cancer | Inhalation: < 10⁻⁶ | 100.0 |
| Cancer-risk interval frequency | IVBA-adjusted ingestion; total As for dermal/inhalation | Adult | Cancer | Inhalation: 10⁻⁶ to < 10⁻⁵ | 0.0 |
| Cancer-risk interval frequency | IVBA-adjusted ingestion; total As for dermal/inhalation | Adult | Cancer | Inhalation: 10⁻⁵ to < 10⁻⁴ | 0.0 |
| Cancer-risk interval frequency | IVBA-adjusted ingestion; total As for dermal/inhalation | Adult | Cancer | Inhalation: ≥ 10⁻⁴ | 0.0 |
| Cancer-risk interval frequency | IVBA-adjusted ingestion; total As for dermal/inhalation | Adult | Cancer | Multi-pathway: < 10⁻⁶ | 0.0 |
| Cancer-risk interval frequency | IVBA-adjusted ingestion; total As for dermal/inhalation | Adult | Cancer | Multi-pathway: 10⁻⁶ to < 10⁻⁵ | 0.0 |
| Cancer-risk interval frequency | IVBA-adjusted ingestion; total As for dermal/inhalation | Adult | Cancer | Multi-pathway: 10⁻⁵ to < 10⁻⁴ | 79.7 |
| Cancer-risk interval frequency | IVBA-adjusted ingestion; total As for dermal/inhalation | Adult | Cancer | Multi-pathway: ≥ 10⁻⁴ | 20.3 |

Table S11. Percentage contribution of each exposure pathway to total non-cancer and cancer risk under the total-As scenario.

| **Receptor** | **Risk endpoint** | **Exposure pathway** | **Mean contribution (%)** | **Median contribution (%)** | **P05 (%)** | **P95 (%)** |
| --- | --- | --- | --- | --- | --- | --- |
| Child | HI | Ingestion | 60.7 | 62.2 | 30.0 | 86.3 |
| Child | HI | Dermal contact | 39.3 | 37.8 | 13.7 | 69.9 |
| Child | HI | Inhalation | 0.018 | 0.016 | 0.006 | 0.039 |
| Child | CR_total_ | Ingestion | 60.7 | 62.2 | 30.0 | 86.3 |
| Child | CR_total_ | Dermal contact | 39.3 | 37.8 | 13.7 | 70.0 |
| Child | CR_total_ | Inhalation | 0.008 | 0.007 | 0.003 | 0.016 |
| Adult | HI | Ingestion | 38.6 | 35.9 | 8.4 | 77.6 |
| Adult | HI | Dermal contact | 61.3 | 64.0 | 22.4 | 91.5 |
| Adult | HI | Inhalation | 0.057 | 0.058 | 0.021 | 0.087 |
| Adult | CR_total_ | Ingestion | 38.6 | 35.9 | 8.4 | 77.6 |
| Adult | CR_total_ | Dermal contact | 61.3 | 64.0 | 22.4 | 91.5 |
| Adult | CR_total_ | Inhalation | 0.023 | 0.024 | 0.009 | 0.036 |

Figure S1. Relationship between total As concentration and IVBA. The solid line shows the fitted linear regression, and the shaded band represents the 95% confidence interval. The regression equation, coefficient of determination (R²) and p-value are displayed in the figure.
